# Towards Universal Voluntary HIV Testing and Counselling: A Systematic Review and Meta-Analysis of Community-Based Approaches

**DOI:** 10.1371/journal.pmed.1001496

**Published:** 2013-08-13

**Authors:** Amitabh B. Suthar, Nathan Ford, Pamela J. Bachanas, Vincent J. Wong, Jay S. Rajan, Alex K. Saltzman, Olawale Ajose, Ade O. Fakoya, Reuben M. Granich, Eyerusalem K. Negussie, Rachel C. Baggaley

**Affiliations:** 1 Department of HIV/AIDS, World Health Organization, Geneva, Switzerland; 2 Global AIDS Program, Centers for Disease Control and Prevention, Atlanta, Georgia, United States of America; 3 Office of HIV/AIDS, United States Agency for International Development, Washington, District of Columbia, United States of America; 4 School of Medicine, University of California at San Francisco, San Francisco, California, United States of America; 5 School of Medicine, Cornell University, New York, New York, United States of America; 6 Clinton Health Access Initiative, Boston, Massachusetts, United States of America; 7 The Global Fund to Fight AIDS, Tuberculosis and Malaria, Geneva, Switzerland; 8 Joint United Nations Programme on HIV/AIDS, Geneva, Switzerland; Centers for Disease Control and Prevention, United States of America

## Abstract

**Background:**

Effective national and global HIV responses require a significant expansion of HIV testing and counselling (HTC) to expand access to prevention and care. Facility-based HTC, while essential, is unlikely to meet national and global targets on its own. This article systematically reviews the evidence for community-based HTC.

**Methods and Findings:**

PubMed was searched on 4 March 2013, clinical trial registries were searched on 3 September 2012, and Embase and the World Health Organization Global Index Medicus were searched on 10 April 2012 for studies including community-based HTC (i.e., HTC outside of health facilities). Randomised controlled trials, and observational studies were eligible if they included a community-based testing approach and reported one or more of the following outcomes: uptake, proportion receiving their first HIV test, CD4 value at diagnosis, linkage to care, HIV positivity rate, HTC coverage, HIV incidence, or cost per person tested (outcomes are defined fully in the text). The following community-based HTC approaches were reviewed: (1) door-to-door testing (systematically offering HTC to homes in a catchment area), (2) mobile testing for the general population (offering HTC via a mobile HTC service), (3) index testing (offering HTC to household members of people with HIV and persons who may have been exposed to HIV), (4) mobile testing for men who have sex with men, (5) mobile testing for people who inject drugs, (6) mobile testing for female sex workers, (7) mobile testing for adolescents, (8) self-testing, (9) workplace HTC, (10) church-based HTC, and (11) school-based HTC. The Newcastle-Ottawa Quality Assessment Scale and the Cochrane Collaboration's “risk of bias” tool were used to assess the risk of bias in studies with a comparator arm included in pooled estimates.

117 studies, including 864,651 participants completing HTC, met the inclusion criteria. The percentage of people offered community-based HTC who accepted HTC was as follows: index testing, 88% of 12,052 participants; self-testing, 87% of 1,839 participants; mobile testing, 87% of 79,475 participants; door-to-door testing, 80% of 555,267 participants; workplace testing, 67% of 62,406 participants; and school-based testing, 62% of 2,593 participants. Mobile HTC uptake among key populations (men who have sex with men, people who inject drugs, female sex workers, and adolescents) ranged from 9% to 100% (among 41,110 participants across studies), with heterogeneity related to how testing was offered. Community-based approaches increased HTC uptake (relative risk [RR] 10.65, 95% confidence interval [CI] 6.27–18.08), the proportion of first-time testers (RR 1.23, 95% CI 1.06–1.42), and the proportion of participants with CD4 counts above 350 cells/µl (RR 1.42, 95% CI 1.16–1.74), and obtained a lower positivity rate (RR 0.59, 95% CI 0.37–0.96), relative to facility-based approaches. 80% (95% CI 75%–85%) of 5,832 community-based HTC participants obtained a CD4 measurement following HIV diagnosis, and 73% (95% CI 61%–85%) of 527 community-based HTC participants initiated antiretroviral therapy following a CD4 measurement indicating eligibility. The data on linking participants without HIV to prevention services were limited. In low- and middle-income countries, the cost per person tested ranged from US$2–US$126. At the population level, community-based HTC increased HTC coverage (RR 7.07, 95% CI 3.52–14.22) and reduced HIV incidence (RR 0.86, 95% CI 0.73–1.02), although the incidence reduction lacked statistical significance. No studies reported any harm arising as a result of having been tested.

**Conclusions:**

Community-based HTC achieved high rates of HTC uptake, reached people with high CD4 counts, and linked people to care. It also obtained a lower HIV positivity rate relative to facility-based approaches. Further research is needed to further improve acceptability of community-based HTC for key populations. HIV programmes should offer community-based HTC linked to prevention and care, in addition to facility-based HTC, to support increased access to HIV prevention, care, and treatment.

**Review Registration:**

International Prospective Register of Systematic Reviews CRD42012002554

*Please see later in the article for the Editors' Summary*

## Abstract 2

In a systematic review and meta-analysis, Amitabh Suthar and colleagues describe the evidence base for different HIV testing and counseling services provided outside of health facilities.

*Please see later in the article for the Editors' Summary*

## Editors' Summary

**Background:**

Three decades into the AIDS epidemic, about 34 million people (most living in resource-limited countries) are infected with HIV, the virus that causes AIDS. Every year another 2.2 million people become infected with HIV, usually through unprotected sex with an infected partner, and about 1.7 million people die. Infection with HIV, which gradually destroys the CD4 lymphocytes and other immune system cells that provide protection from life-threatening infections, is usually diagnosed by looking for antibodies to HIV in the blood or saliva. Disease progression is subsequently monitored in HIV-positive individuals by counting the CD4 cells in their blood. Initiation of antiretroviral drug therapy—a combination of drugs that keeps HIV replication in check but that does not cure the infection—is recommended when an individual's CD4 count falls below 500 cells/µl of blood or when he or she develops signs of severe or advanced disease, such as unusual infections.

**Why Was This Study Done?:**

As part of intensified efforts to eliminate HIV/AIDS, United Nations member states recently set several HIV-related targets to be achieved by 2015, including reduced transmission of HIV and increased delivery of antiretroviral therapy. These targets can only be achieved if there is a large expansion in HIV testing and counseling (HTC) and increased access to HIV prevention and care services. The World Health Organization currently recommends that everyone attending a healthcare facility in regions where there is a generalized HIV epidemic (defined as when 1% or more of the general population is HIV-positive) should be offered HTC. However, many people rarely visit healthcare facilities, and others refuse “facility-based” HTC because they fear stigmatization and discrimination. Thus, facility-based HTC alone is unlikely to be sufficient to enable national and global HIV targets to be reached. In this systematic review and meta-analysis, the researchers evaluate the performance of community-based HTC approaches such as index testing (offering HTC to the sexual and injecting partners and household members of people with HIV), mobile testing (offering HTC through a service that visits shopping centers and other public facilities), and door-to-door testing (systematically offering HTC to homes in a catchment area). A systematic review uses predefined criteria to identify all the research on a given topic; meta-analysis combines the results of several studies.

**What Did the Researchers Do and Find?:**

The researchers identified 117 studies (most undertaken in Africa and North America) involving 864,651 participants that evaluated community-based HTC approaches. Among these studies, the percentage of people offered community-based HTC who accepted it (HTC uptake) was 88% for index testing, 87% for self-testing, 80% for door-to-door testing, 67% for workplace testing, and 62% for school-based testing. Compared to facility-based approaches, community-based approaches increased the chances of an individual's CD4 count being above 350 cells/µl at diagnosis (an important observation because early diagnosis improves subsequent outcomes) but had a lower positivity rate, possibly because people with symptoms of HIV are more likely to visit healthcare facilities than healthy individuals. Importantly, 80% of participants in the community-based HTC studies had their CD4 count measured after HIV diagnosis, and 73% of the participants initiated antiretroviral therapy after their CD4 count fell below national eligibility criteria; both these observations suggest that community-based HTC successfully linked people to care. Finally, offering community-based HTC approaches in addition to facility-based approaches increased HTC coverage seven-fold at the population level.

**What Do These Findings Mean?:**

These findings show that community-based HTC can achieve high HTC uptake rates and can reach HIV-positive individuals earlier, when they still have high CD4 counts. Importantly, they also suggest that the level of linkage to care of community-based HTC is similar to that of facility-based HTC. Although the lower positivity rate of community-based HTC approaches means that more people need to be tested with these approaches than with facility-based HTC to identify the same number of HIV-positive individuals, this downside of community-based HTC is likely to be offset by the earlier identification of HIV-positive individuals, which should improve life expectancy and reduce HIV transmission at the population level. Although further studies are needed to evaluate community-based HTC in other regions of the world, these findings suggest that offering community-based HTC in HIV programs in addition to facility-based testing should support the increased access to HIV prevention and care that is required for the intensification of HIV/AIDS elimination efforts.

**Additional Information:**

Please access these websites via the online version of this summary at http://dx.doi.org/10.1371/journal.pmed.1001496.

- The World Health Organization provides information on all aspects of HIV/AIDS, including information on counseling and testing (in several languages)
- Information is available from the US National Institute of Allergy and Infectious Diseases on HIV infection and AIDS
- NAM/aidsmap provides basic information about HIV/AIDS and summaries of recent research findings on HIV care and treatment
- Information is available from Avert, an international AIDS charity, on many aspects of HIV/AIDS, including information on the global HIV/AIDS epidemic, on HIV testing, and on HIV transmission and testing (in English and Spanish)
- The UK National Health Service Choices website provides information (including personal stories) about HIV and AIDS
- The *World AIDS Day Report 2012* provides up-to-date information about the AIDS epidemic and efforts to halt it
- Patient stories about living with HIV/AIDS are available through Avert; the nonprofit website Healthtalkonline also provides personal stories about living with HIV, including stories about getting a diagnosis

## Introduction

HIV is a leading cause of morbidity and mortality globally [1]. Despite considerable progress in controlling the epidemic, there were approximately 2.2 million new HIV infections, 1.7 million HIV-related deaths, and 34.2 million people with HIV worldwide in 2011; 1.5 million of these new HIV infections, 1.2 million of the HIV deaths, and 23.5 million of the people living with HIV were in Africa [2]. Given the urgency to act on the epidemic, all United Nations member states agreed to achieve the following HIV targets by 2015: (1) reduce sexual and parenteral HIV transmission by 50%, (2) eliminate vertical HIV transmission, (3) reduce tuberculosis deaths among people with HIV by 50%, and (4) deliver antiretroviral therapy (ART) to 15 million people [3]. Achieving these targets will require people at risk of HIV to learn their status and link to prevention and care services.

In an effort to expand access to prevention and care services, World Health Organization (WHO) guidelines recommend provider-initiated HIV testing and counselling (HTC) for all people seen in all health facilities in generalised epidemics (i.e., antenatal HIV prevalence ≥1%) and in specific facilities in concentrated epidemics [4]. While provider-initiated HTC programmes have been successful in identifying previously undiagnosed individuals in generalised epidemics, they may not reach all people at risk of HIV acquisition [5],[6]. Indeed, the latest Demographic and Health Surveys from 29 sub-Saharan African countries, representing approximately half of the global burden of HIV, indicate that only 15% of adults received results from an HIV test in the previous year [7]. This low coverage is recognised as a critical barrier to scaling up HIV prevention and care interventions. Furthermore, people living with HIV are often diagnosed late in the course of their disease, resulting in avoidable morbidity, mortality, and transmission of the virus. [8].

The reasons for the current low coverage of HTC are various and include service, patient, and demographic barriers [9],[10]. For example, in generalised epidemics women have higher rates of testing than men and adolescents, perhaps because of their contact with reproductive and antenatal health services [7]. Implementation of provider-initiated HTC guidance remains a priority for countries. However, because many people have limited contact with healthcare providers, HTC provision in health facilities alone is insufficient to achieve national and global targets. Although previous research has reviewed home-based HTC [11],[12], the impact of all community-based HTC approaches has not been systematically reviewed. The objective of this study was to systematically review all community-based HTC approaches to inform global and national HIV programming.

## Methods

### Conduct of Systematic Review

This systematic review was conducted in accordance with the PRISMA statement using a pre-defined protocol (International Prospective Register of Systematic Reviews identification number: CRD42012002554; Text S1 and Protocol S1) [13],[14]. The PubMed database was searched on 4 March 2013, and Embase and WHO Global Index Medicus were systematically searched on 10 April 2012, without language, geographic, publication, date, or any other restrictions. In addition, the WHO International Clinical Trials Registry Platform, the Cochrane Central Register of Controlled Trials, the International Standard Randomised Controlled Trial Number Register, and ClinicalTrials.gov were systematically searched without language, publication, or date restrictions on 3 September 2012. Experts in the field were contacted to identify unpublished research and ongoing studies, and bibliographies of relevant studies were screened.

### Study Definitions

Community-based HTC was defined as HTC outside of health facilities. Facility-based HTC approaches were defined as those in healthcare sites (e.g., health facilities, hospitals, and fixed, stand-alone voluntary counselling and testing sites). Eleven different community-based HTC approaches were reviewed in this study: (1) door-to-door testing (systematically offering HTC to homes in a catchment area), (2) mobile testing for the general population (offering HTC via a mobile HTC service in areas visited by the general public, such as shopping centres, transport hubs, or roadside restaurants), (3) index testing (offering HTC to household members of people with HIV and persons who may have been exposed to HIV such as spouses, sexual partners, or children of people with HIV); (4) mobile testing for men who have sex with men (MSM), (5) mobile testing for people who inject drugs (PWID), (6) mobile testing for female sex workers (FSW), (7) mobile testing for adolescents, (8) self-testing, (9) workplace HTC, (10) church-based HTC, and (11) school-based HTC.

Several outcomes were analysed in this study. Uptake was calculated by dividing the number of individuals accepting HTC by the number of individuals offered HTC. The proportion of first-time testers was calculated by dividing the number of people reporting receiving their first HIV test by the total number of people tested. The proportion of participants with a CD4 count greater than 350 cells/µl was calculated among participants with HIV who had their CD4 count measured. Two steps of the retention continuum were assessed: (1) CD4 measurement (among all participants found to have HIV) and (2) initiation of ART (among participants eligible per national guidelines). In studies with a comparator arm, the HIV positivity rate was calculated by dividing the number of individuals found to be HIV positive by the number of individuals tested. HTC coverage was calculated by dividing the number of people tested by the total number of people living in the catchment area for the community-based HTC approach. HIV incidence was calculated by dividing the risk of infection in communities with access to community- and facility-based HTC by the risk of infection in communities with access to only facility-based HTC. Some of the outcomes were not independent. For example, the number of people tested was the denominator for the HIV positivity rate and first-time testers and also the numerator for HTC coverage. Moreover, the number of people living with HIV was the numerator for the HIV positivity rate and also the denominator for calculating the first step of the retention continuum (CD4 measurement). The cost per person tested was approximated by dividing the economic costs incurred during HTC in studies by the total number of people tested. Costs were adjusted for inflation from the year the costs were estimated to 2012 United States dollars using the US Bureau of Labor Statistics' inflation calculator [15].

### Search Strategy and Selection Criteria

The search strategies (Table S1) were designed with the assistance of a librarian to identify studies including community-based HTC. Following recommendations from PRISMA, eligibility criteria were based on key study characteristics: population, intervention, comparator, outcome, and design [13]. Specifically, studies were included when (1) the study population included people in generalised, concentrated, or low-level HIV epidemics; (2) the intervention was community-based HTC offered in combination with a background of facility-based HTC; (3) the comparator was facility-based HTC; (4) the outcome(s) included uptake, proportion of people reporting receiving their first HIV test, CD4 value at diagnosis, rates of linkage to care, HIV positivity rate, HTC coverage, HIV incidence, or cost per person tested; and (5) the study design was a randomised trial or observational cohort study. Given the lack of comparative studies for community-based HTC, studies without a comparator arm were also included if they met the remaining eligibility criteria.

A. B. S., N. F., and O. A. independently screened the abstracts of all articles identified via the literature database searches and then compared the full texts of all articles selected during screening against the inclusion criteria. Disagreements were resolved by discussion. J. S. R. and A. K. S. repeated the same process for the clinical trial registries.

### Data Extraction

A. B. S., J. S. R., and A. K. S. completed the data extraction of characteristics of study participants, community-based testing approaches, outcomes, and quality assessment using a standardised extraction form.

### Quality Assessment

The Newcastle-Ottawa Quality Assessment Scale was used to assess bias in studies with a comparator arm included in pooled analyses [16]. This scale rates studies based on eight criteria in three sources of bias. We modified this scale to remove one criterion, demonstration that the outcome of interest was not present at the start of study, since a previous HIV test may not affect all the outcomes analysed in this article. The Cochrane Collaboration's “risk of bias” tool was used to assess bias in randomised trials with a comparator arm [17].

### Statistical Analyses

Outcome proportions from studies meeting inclusion criteria were stabilised using the Freeman-Tukey-type arcsine square-root transformation and then pooled to summarise the proportion of participants who (1) accepted different community-based HTC approaches, (2) reported receiving their first HIV test, (3) had CD4 counts measured after diagnosis, (4) were diagnosed with HIV with a CD4 count above 350 cells/µl, and (5) initiated ART after their CD4 count indicated they were eligible for treatment [18],[19]. Pooled relative risks (RRs) were used to compare participants of community- and facility-based HTC with respect to uptake, proportion of first-time testers, the HIV positivity rate, proportion with CD4 counts above 350 cells/µl, and HTC coverage. Random-effects models were used for all analyses. Given the differences in HIV epidemiology, sexual mixing patterns, transmission factors, and healthcare utilisation rates for key populations, key population outcome data were reported individually and not pooled. *I* ^2^ statistics were used to measure heterogeneity [20]. *I* ^2^ statistics near 25% indicate low heterogeneity, values near 50% indicate moderate heterogeneity, and those above 75% indicate high heterogeneity [21]. All analyses were completed in STATA version 12.0.

## Results

### Search Results

**Figure 1:**
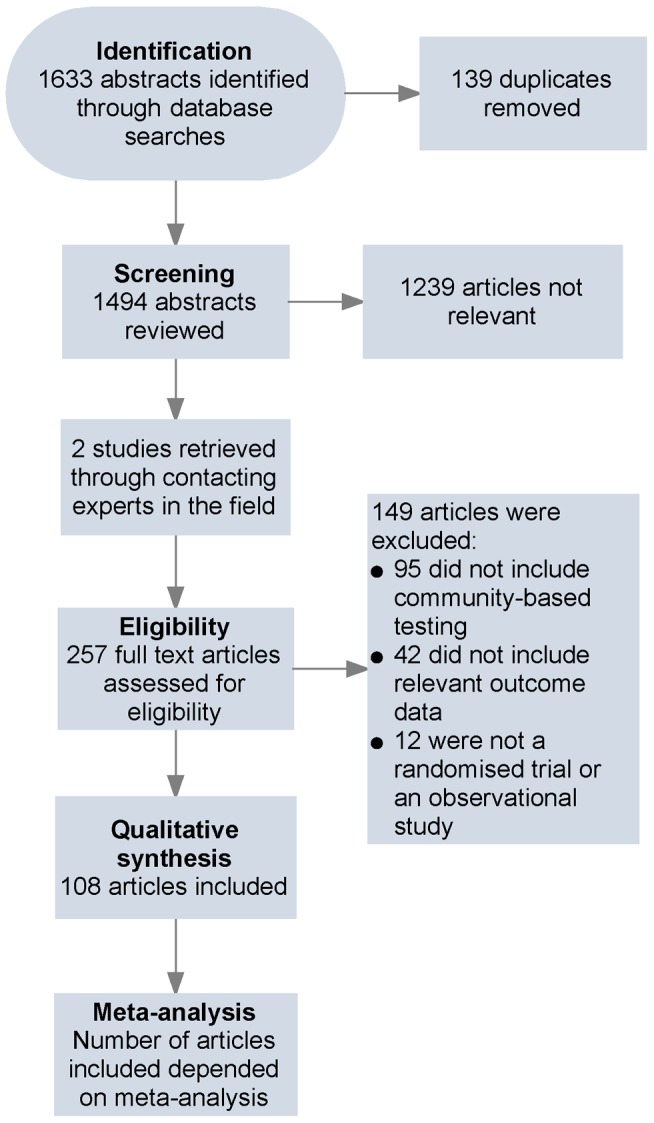
Flow of information through different phases of the review.

108 articles, describing studies conducted from 1987 to 2012 and including 864,651 participants completing HTC, met the eligibility criteria (Table 1; Figure 1). Two articles were randomised trials [22],[23], and the rest were observational in design. Data from one multi-centre cluster-randomised trial were stratified into three studies (based on the country where the testing was offered) [23], data from three articles were stratified based on the year community-based HTC was offered [24]–[26], and data from four articles were stratified based on the community-based HTC approach used [27]–[30]. Given that 108 articles provided data from 108 studies and there were nine additional studies after stratification, there were a total of 117 studies included (Table S4). 76 studies were from Africa, 28 were from North America (excluding Central America), six were from Asia, four were from Central and South America, three were from Europe, and one was from Australia. The clinical trial registers identified ten ongoing trials: one on index testing [31], one on mobile testing [32], five on door-to-door testing [33]–[37], one on self-testing [38], and two on community-based testing for key populations [39],[40].

**Table 1:** Summary of study participants and methods.

Testing Model	Number of Studies	Total Number Tested	Median Year Study Conducted (IQR)^a^	Number of Males (Percent)^b^	Number of Studies with a Demand Creation Component (Percent)	Number of Studies Providing Incentives (Percent)	Number of Studies with a Multi-Disease Component (Percent)	Number of Studies Linking People with HIV to Care (Percent)
Index	8	12,400	2005 (2004 to 2006)	5,556 (45.3)	0 (0)	1 (12.5)	2 (25.0)	5 (62.5)
Door-to-door	33	595,389	2008 (2004 to 2009)	247,439 (45.9)	11 (33.3)	2 (6.1)	8 (24.2)	19 (57.6)
Mobile	34	193,602	2008 (2005 to 2009)	86,989 (44.9)	20 (60.6)	7 (20.6)	15 (44.1)	16 (47.1)
Key populations	29	41,451	2005 (2002 to 2008)	12,866 (61.9)	10 (34.5)	15 (51.7)	9 (31.0)	16 (55.2)
Self	3	1,779	2006 (2002 to 2008)	1,113 (62.6)	1 (33.3)	2 (66.7)	0 (0.0)	1 (33.3)
Workplace	6	17,352	2004 (2003 to 2009)	9,817 (67.0)	2 (33.3)	1 (16.7)	4 (66.7)	3 (50.0)
School	4	2,678	2009 (2005 to 2009)	957 (42.2)	2 (50.0)	0 (0.0)	2 (50.0)	3 (75.0)

a The midpoint was used for studies that took place over several years.

b Among studies that included gender data.

IQR, interquartile range.

The percentage of participants who were male was 45.3% for index testing, 45.9% for door-to-door testing, 44.9% for mobile testing, 62.6% for self-testing, 67.0% for workplace testing, and 42.2% for school-based testing (Table 1). Excluding studies including only MSM or only FSW, 62.9% of testers were male in mobile testing for key populations (Table 1). Population-level HTC efforts found that implementation of community-based HTC increases the number of couples receiving testing (Table 2).

**Table 2:** Percentage of clients received as couples in community-wide testing efforts.

Study (Testing Approach)	Country	Year	Number Tested as a Couple	Number Tested	Percent Tested as a Couple
Sweat (facility-based) [23]	Thailand	2007	1,472	2,721	54.1%
Sweat (mobile) [23]	Thailand	2007	2,574	10,464	24.6%
Tumwesigye (door-to-door) [74]	Uganda	2007	35,634	264,966	13.4%
Sweat (facility-based) [23]	Zimbabwe	2007	61	610	10.0%
Naik (door-to-door) [70]	South Africa	2010	458	5,086	9.1%
Lugada (mobile) [56]	Kenya	2008	3,296	47,173	7.0%
Sweat (facility-based) [23]	Tanzania	2007	24	685	3.5%
Sweat (mobile) [23]	Zimbabwe	2007	223	6,579	3.4%
Sweat (mobile) [23]	Tanzania	2007	54	2,832	1.9%

### Uptake

**Figure 2:**
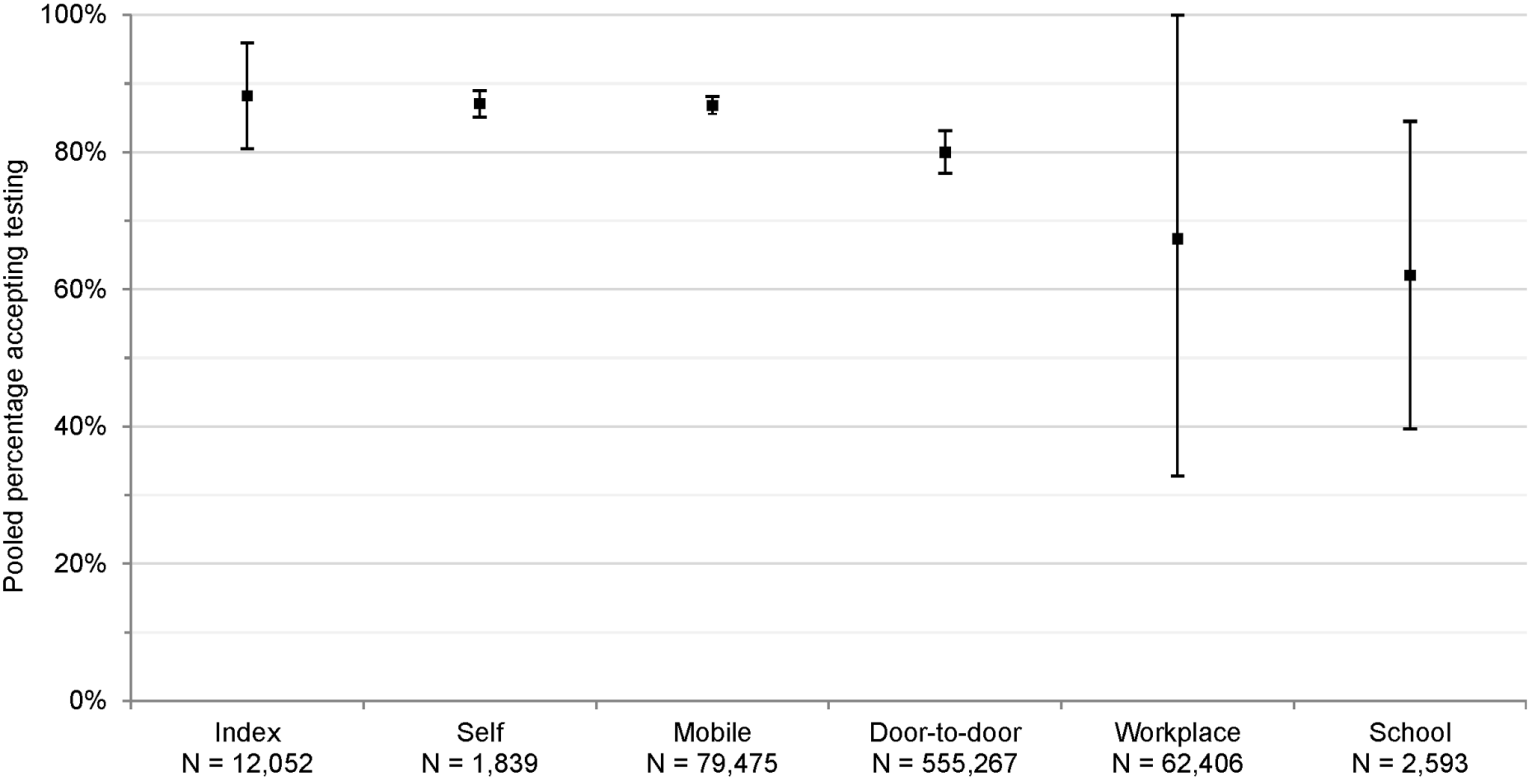
Pooled uptake of community-based HTC approaches. Bars indicate 95% CIs.

**Figure 3:**
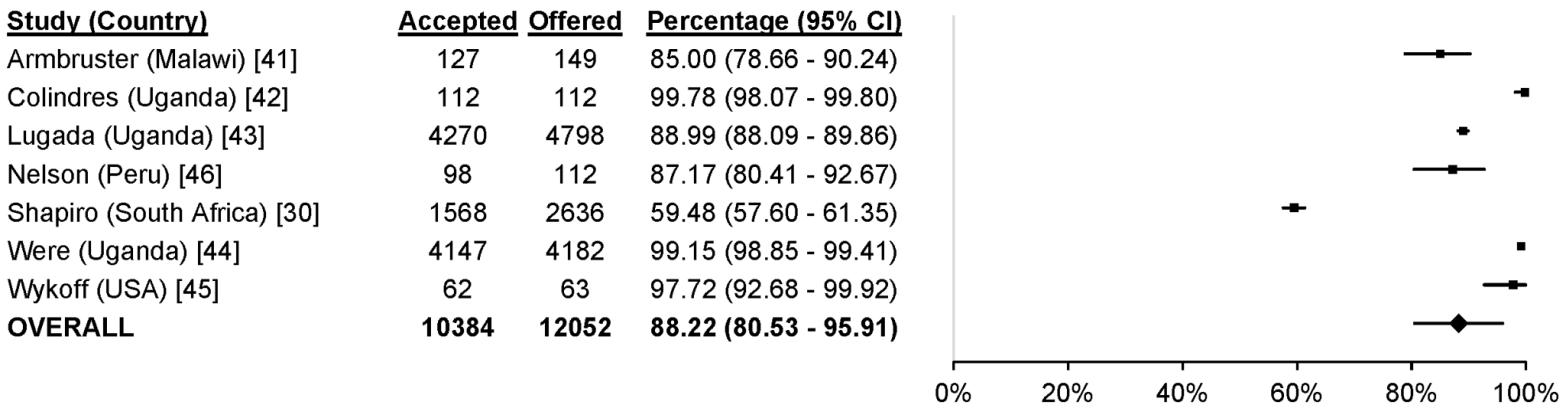
Uptake of index HTC.

**Figure 4:**
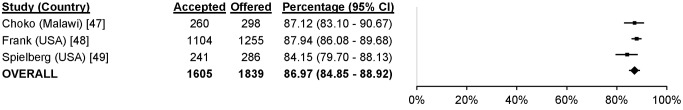
Uptake of self-testing.

**Figure 5:**
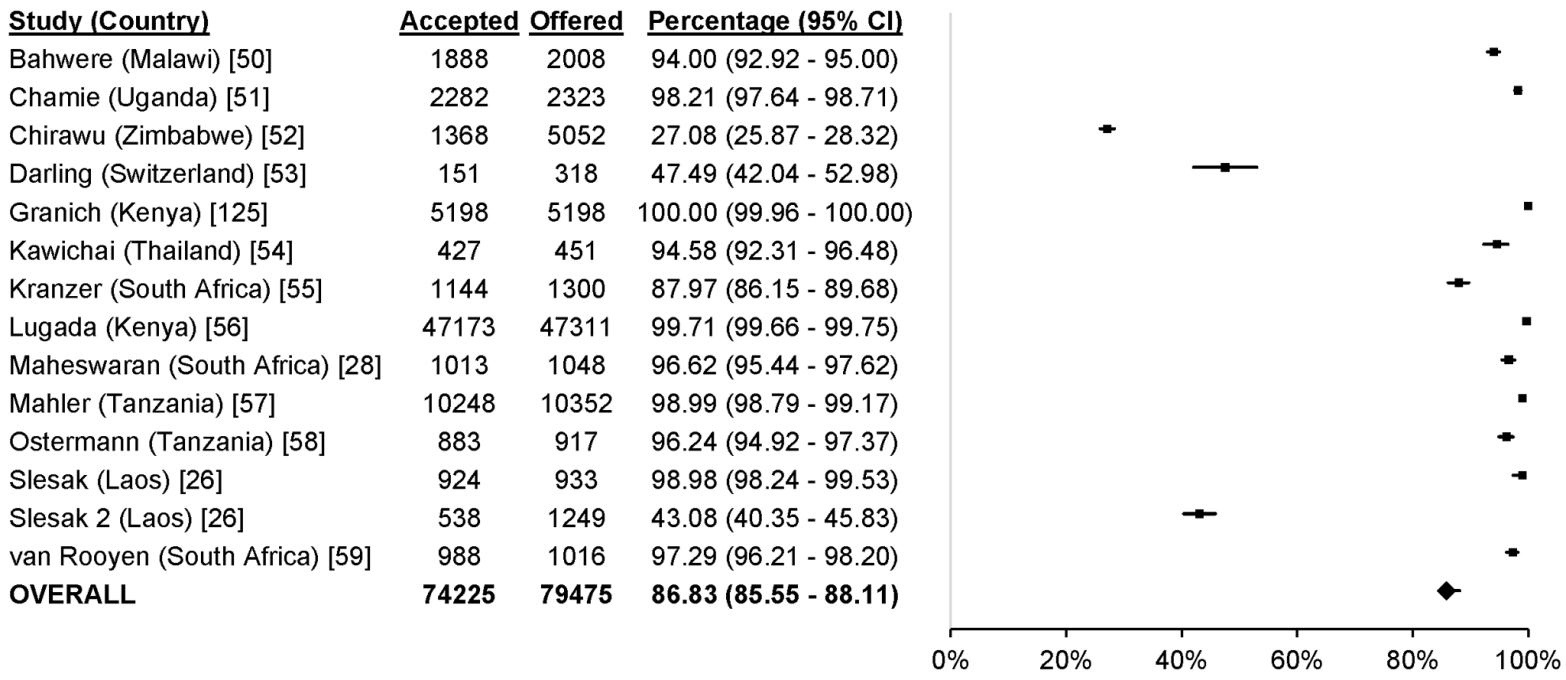
Uptake of mobile HTC.

**Figure 6:**
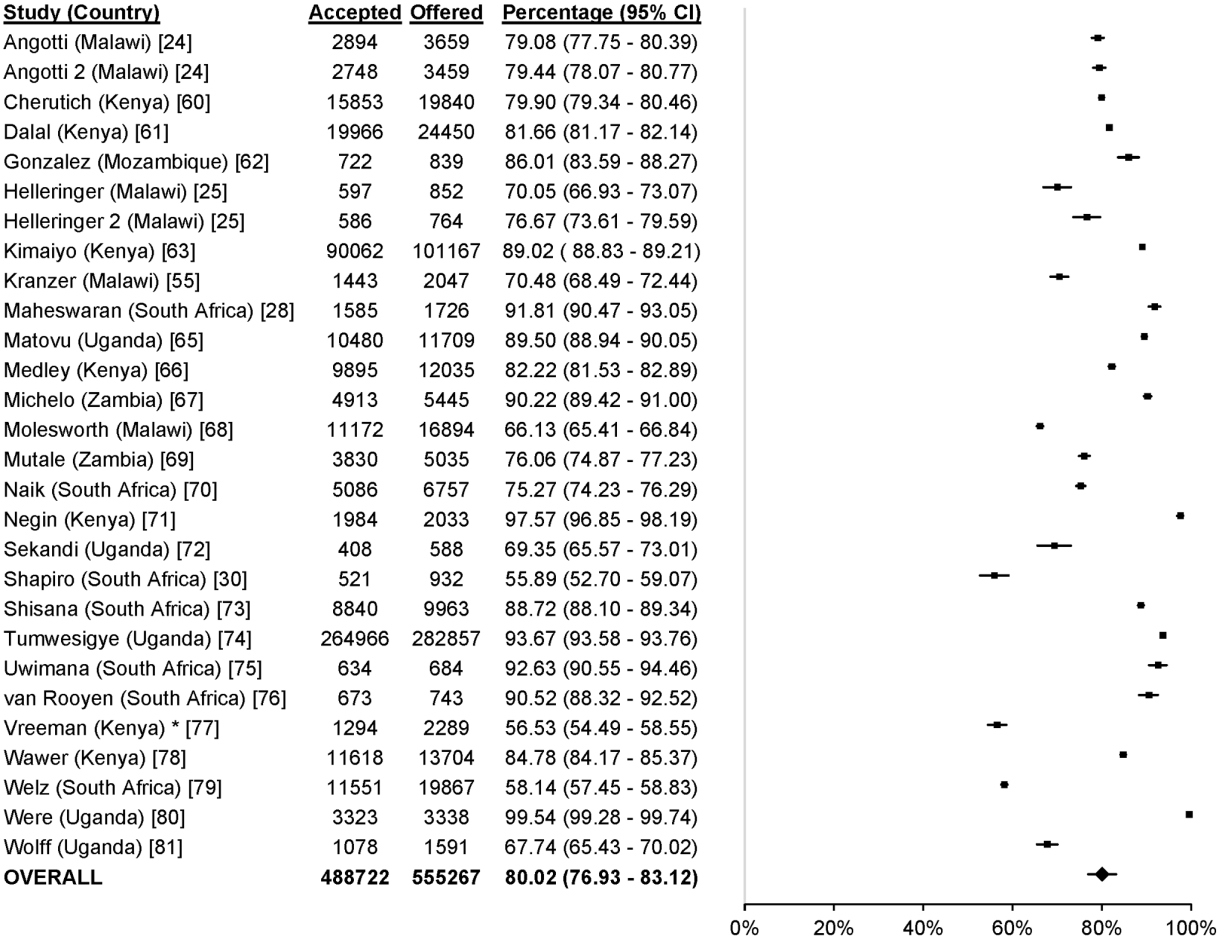
Uptake of door-to-door HTC. Asterisk: data reported were exclusively from children aged 18 mo.–13 y.

**Figure 7:**
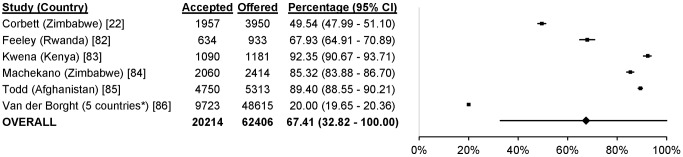
Uptake of workplace HTC. Asterisk: data reported were from the Democratic Republic of Congo, Rwanda, Burundi, Congo, and Nigeria.

**Figure 8:**
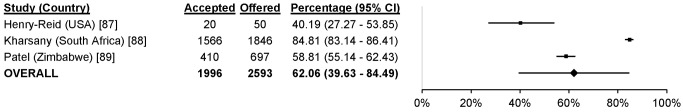
Uptake of school-based HTC.

**Figure 9:**
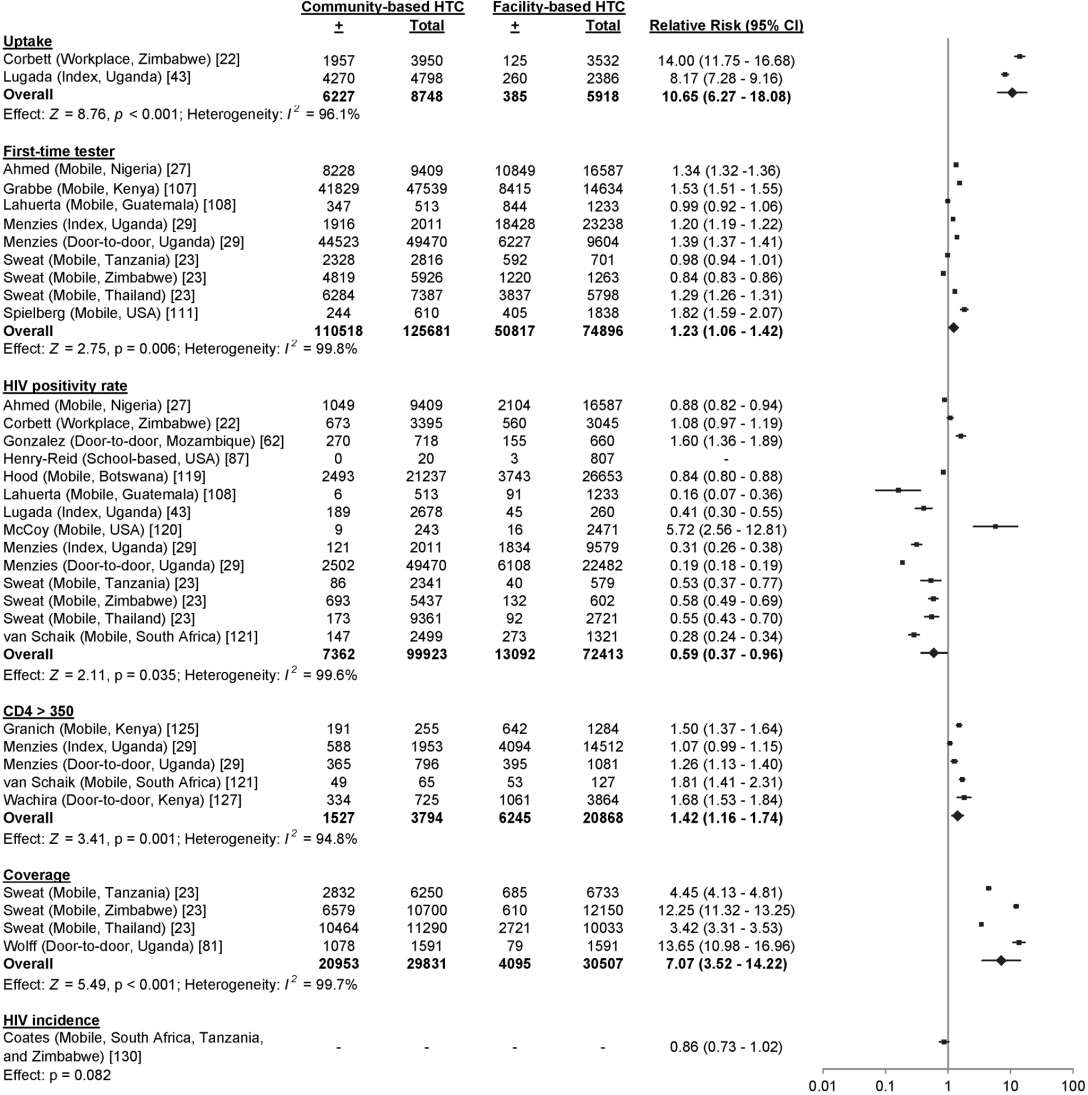
Pooled relative risks of community-based HTC versus facility-based HTC. The numerator for all RRs was the risk of an outcome in community-based testing, while the denominator was the risk of an outcome in facility-based testing.

61 studies reported uptake of different community-based testing approaches among 713,632 participants: seven studies evaluated index testing among 12,052 participants [29],[30],[41]–[46], three evaluated self-testing among 1,839 participants [47]–[49], 14 evaluated mobile HTC among 79,475 participants [26],[28],[50]–[59], 28 evaluated door-to-door testing among 555,267 participants [24],[25],[28],[30],[60]–[81], six evaluated workplace HTC among 62,406 participants [22],[82]–[86], and three evaluated school-based HTC among 2,593 participants [87]–[89] (Figure 2). The percentage of participants accepting HTC was 88.2% for index testing (95% confidence interval [CI] 80.5%–95.9%; *I* ^2^ 99.7%, 95% CI 99.7%–99.8%; Figure 3), 87.1% for self-testing (95% CI 85.1%–89.0%; *I* ^2^ 28.8%, 95% CI 0%–92.6%; Figure 4), 86.8% for mobile HTC (95% CI 85.6%–88.1%; *I* ^2^ 99.9%, 95% CI 99.9%–99.9%; Figure 5), 80.0% for door-to-door HTC (95% CI 76.9%–83.1%; *I* ^2^ 99.9%, 95% CI 99.9%–99.9%; Figure 6), 67.4% for workplace HTC (95% CI 32.8%–100.0%; *I* ^2^ 100%, 100.0%–100.0%; Figure 7), and 62.1% for school-based HTC (95% CI 39.6%–84.5%; *I* ^2^ 99.0%, 95% CI 98.5%–99.4%; Figure 8). Uptake was higher in community-based HTC compared to providing vouchers to participants for facility-based HTC (RR 10.65, 95% CI 6.27–18.08; *I* ^2^ 96.1%; Figure 9) [22],[43].

**Figure 10:**
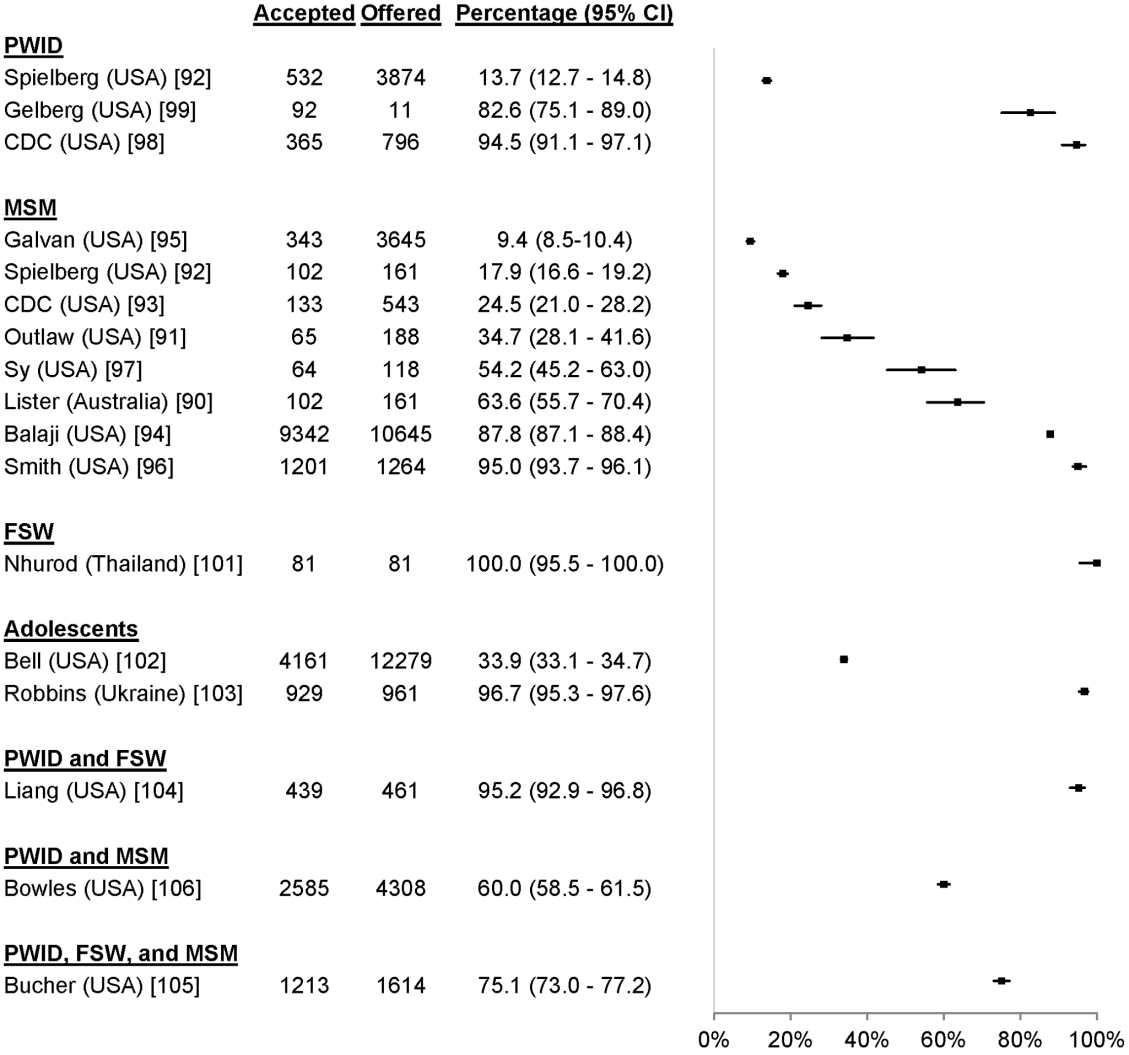
Uptake of community-based HTC approaches among key populations.

**Figure 11:**
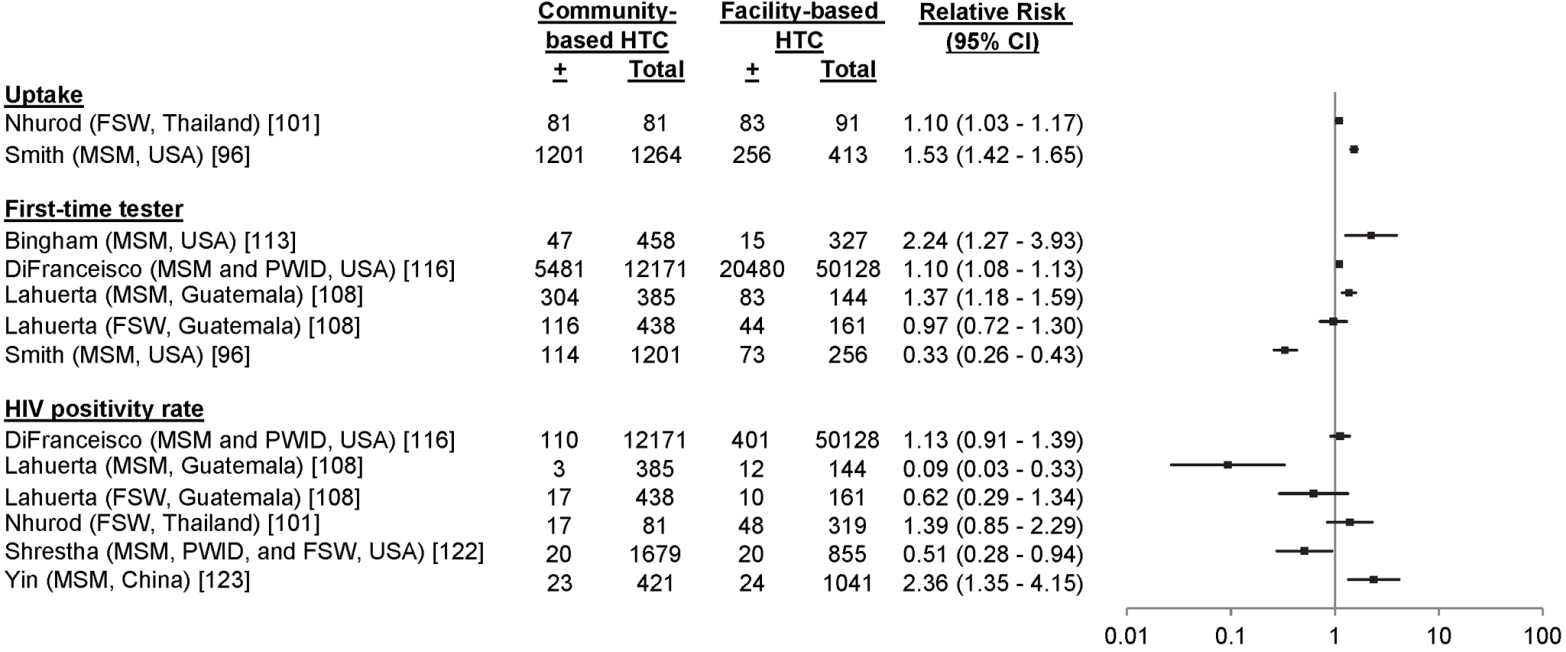
Relative risks of community-based HTC versus facility-based HTC among key populations. The numerator for all RRs was the risk of an outcome in community-based testing, while the denominator was the risk of an outcome in facility-based testing.

19 studies reported uptake among 41,110 participants in key populations, including 16,725 MSM [90]–[97], 4,681 PWID [92],[98]–[100], 81 FSW [101], 13,240 adolescents [102],[103], and 6,383 individuals from combinations of key populations. The percentage accepting HTC was 99.7% among FSW, ranged from 13.7% to 94.5% among PWID, ranged from 9.4% to 95.0% among MSM, and ranged from 33.9% to 96.6% among adolescents (Figure 10). One study reported an uptake percentage of 95.2% among PWID and FSW [104], another reported an uptake percentage of 75.1% among PWID, FSW, and MSM [105], and another reported an uptake percentage of 60.0% among PWID and MSM [106]. Uptake was higher for community-based testing than for facility-based testing among FSW (RR 1.10, 95% CI 1.03–1.17) [101] and MSM (RR 1.53, 95% CI 1.42–1.65) [96] (Figure 11).

### First-Time Testers

**Figure 12:**
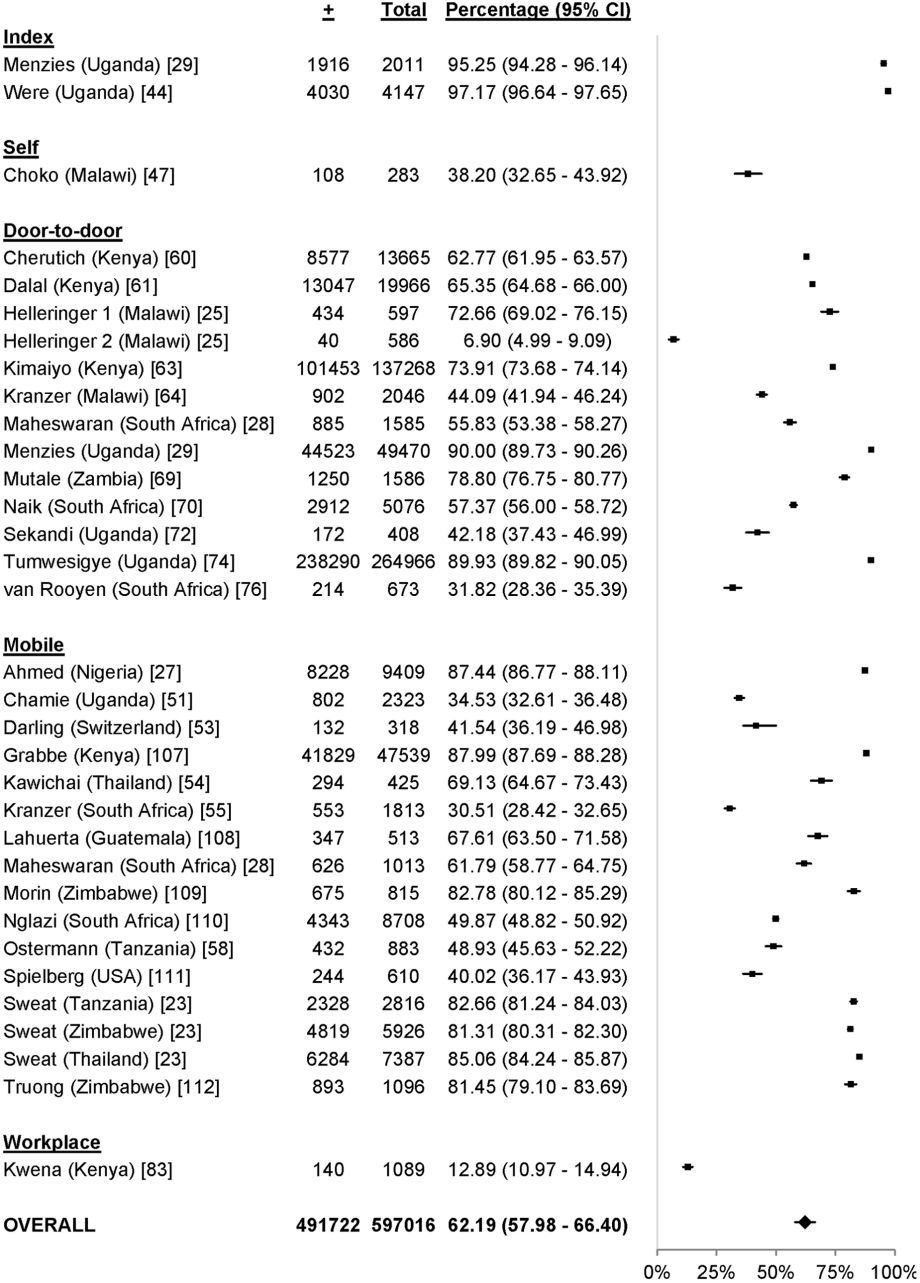
First-time testers in community-based testing approaches.

33 studies reported the HTC history among 597,016 participants in community-based HTC approaches [23],[25],[27]–[29],[44],[47],[51],[53]–[55],[58]–[61],[63],[64],[69],[70],[72],[74],[83],[107]–[112]. 62.2% (95% CI 58.0%–66.4%; *I* ^2^ 99.9%, 95% CI 99.9%–99.9%; Figure 12) of participants at community-based HTC sites reported receiving their first HIV test. In the nine studies with a facility-based comparator arm, a larger proportion of participants reported receiving their first HIV test at community-based HTC than at facility-based HTC (RR 1.23, 95% CI 1.06–1.42; *I* ^2^ 99.8%, 95% CI 99.8%–99.9%; Figure 9) [23],[27],[29],[107],[108],[111].

**Figure 13:**
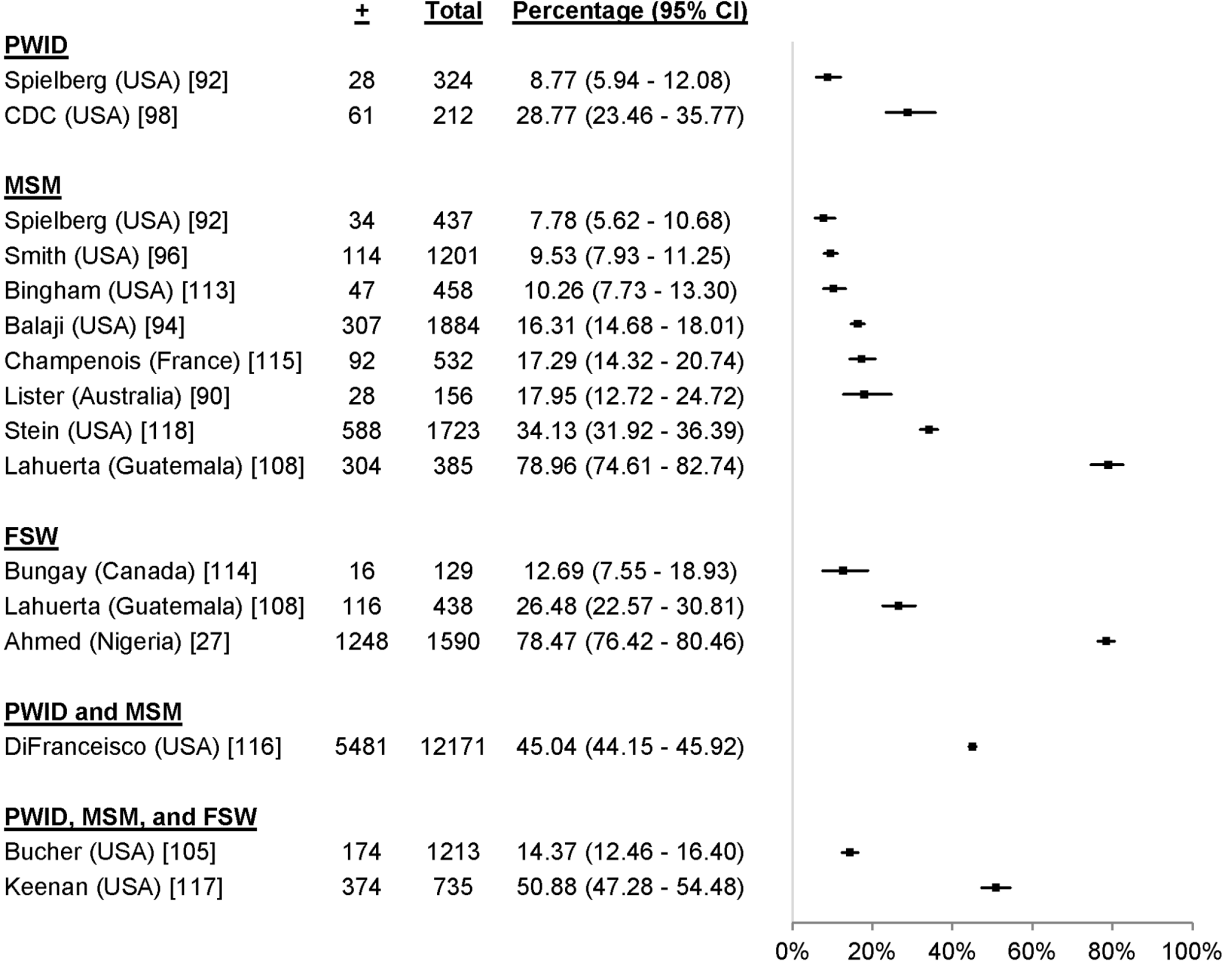
First time testers in community-based testing approaches for key populations.

17 studies reported the HTC history of 25,311 participants from key populations receiving community-based HTC [27],[90],[92],[94],[96],[98],[105],[108],[113]–[118]. 9% to 79% of participants reported receiving their first HIV test (Figure 13). Five of these studies included a facility-based comparator arm (Figure 11). There were more first-time testers in community-based HTC than facility-based HTC for two study populations of MSM (RR 2.24, 95% CI 1.27–3.93 [113] and RR 1.37, 95% CI 1.18–1.59 [108]); however, there were fewer first-time testers in community-based HTC for a different study population of MSM (RR 0.33, 95% CI 0.26–0.43 [96]). There were more first-time testers in community-based HTC than facility-based HTC for a study population including PWID and MSM (RR 1.10, 95% CI 1.08–1.13 [116]); however, there was no difference in the proportion of first-time testers for a study population of FSW (RR 0.97, 95% CI 0.72–1.30 [108]).

### HIV Positivity Rate

14 studies included data on the HIV positivity rate among people testing in community-based approaches relative to people testing in facility-based approaches [22],[23],[27],[29],[43],[62],[87],[108],[119]–[121]. Overall, the HIV positivity rate was lower in community-based approaches relative to facility-based approaches (RR 0.59, 95% CI 0.37–0.96; *I* ^2^ 99.6%, 95% CI 99.6%–99.7%; Figure 9). The median number needed to screen to identify one person with HIV in community- and facility-based HTC was 17 (range 3–86) and 6 (range 4–154), respectively (Table 3). The number needed to screen with community-based testing was highest in settings with a low national HIV prevalence: 54 in Thailand and 86 in Guatemala [23],[108].

**Table 3:** Number needed to screen to identify a person with HIV in studies offering community- and facility-based HTC.

Study (Testing Approach)	Country	Community-Based HTC				Facility-Based HTC			
		Number Positive	Number Tested	Positivity Rate	Number Needed to Screen	Number Positive	Number Tested	Positivity Rate	Number Needed to Screen
Ahmed (mobile) [27]	Nigeria	1,049	9,409	0.11	9	2,104	16,587	0.13	8
Corbett (workplace) [22]	Zimbabwe	673	3,395	0.20	5	560	3045	0.18	5
Gonzalez (door-to-door) [62]	Mozambique	270	718	0.38	3	155	660	0.23	4
Hood (mobile) [119]	Botswana	2,493	21,237	0.12	9	3,743	26,653	0.14	7
Lahuerta (mobile) [108]	Guatemala	6	513	0.01	86	91	1,233	0.07	14
Lugada (index) [43]	Uganda	189	2,678	0.07	14	45	260	0.17	6
McCoy (mobile) [120]	US	9	243	0.04	27	16	2,471	0.01	154
Menzies (index) [29]	Uganda	121	2,011	0.06	17	1,834	9,579	0.19	5
Menzies (door-to-door) [29]	Uganda	2,502	49,470	0.05	20	6,108	22,482	0.27	4
Sweat (mobile) [23]	Tanzania	86	2,341	0.04	27	40	579	0.07	14
Sweat (mobile) [23]	Zimbabwe	693	5,437	0.13	8	132	602	0.22	5
Sweat (mobile) [23]	Thailand	173	9,361	0.02	54	92	2,721	0.03	30
van Schaik (mobile) [121]	South Africa	147	2,499	0.06	17	273	1,321	0.21	5

The Henry-Reid et al. [87] study was excluded since it did not find any people with HIV among the 20 school participants screened.

Six community-based testing studies for key populations included a facility-based comparator arm (Figure 11). Studies including FSW and a combination of PWID and MSM found no difference in the positivity rate for community- versus facility-based approaches (FSW, RR 0.62, 95% CI 0.29–1.34 [108] and RR 1.39, 95% CI 0.85–2.29 [101]; PWID and MSM, RR 1.13, 95% CI 0.91–1.39 [116]). There was a lower positivity rate among a study population of MSM (RR 0.09, 95% CI 0.03–0.33 [108]) and among a study population including PWID, FSW, and MSM (RR 0.51, 95% CI 0.28–0.94 [122]). There was also a higher positivity rate among a study population of MSM (RR 2.37, 95% CI 1.35–4.15 [123]). The number needed to screen to identify one person with HIV varied depending on the key population and study setting (Table 4).

**Table 4:** Number needed to screen to identify a person with HIV in studies offering community- and facility-based HTC to key populations.

Study	Key Population(s)	Country	Community-Based HTC				Facility-Based HTC			
			Number Positive	Number Tested	Positivity Rate	Number Needed to Screen	Number Positive	Number Tested	Positivity Rate	Number Needed to Screen
Lahuerta [108]	MSM	Guatemala	3	385	0.01	128	12	144	0.08	12
Yin [123]	MSM	China	23	421	0.05	18	24	1,041	0.02	43
Lahuerta [108]	FSW	Guatemala	17	438	0.04	26	10	161	0.06	16
Nhurod [101]	FSW	Thailand	17	81	0.21	5	48	319	0.15	7
DiFranceisco [116]	MSM and PWID	US	110	12,171	0.01	111	401	50,128	0.01	125
Shrestha [122]	MSM, PWID, and FSW	US	20	1,679	0.01	84	20	855	0.02	43

### CD4 Counts

**Figure 14:**
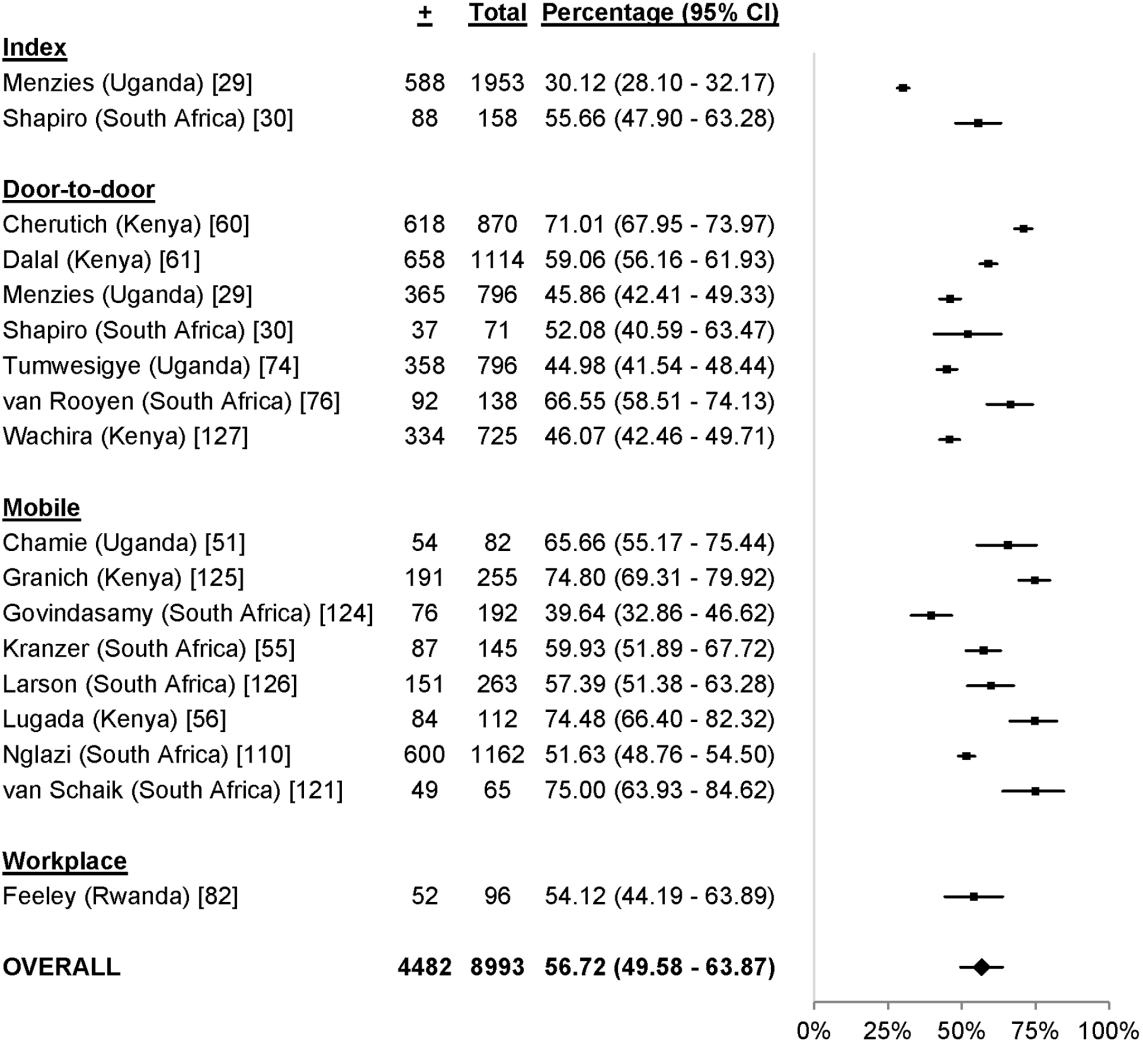
Pooled percentage of community-based HTC participants with CD4 counts above 350 cells/µl.

18 studies reported the CD4 counts of 8,993 participants found to be HIV-positive using point-of-care or standard lab diagnostics [29],[30],[51],[55],[56],[60],[61],[74],[76],[82],[110],[121],[124]–[127]. 56.7% (95% CI 49.6%–63.9%; *I* ^2^ 97.6%, 95% CI 97.0%–98.1%; Figure 14) of participants testing positive had CD4 counts above 350 cells/µl. In the five studies with a facility-based HTC comparator arm, more participants in community-based HTC approaches had CD4 counts above 350 cells/µl than in facility-based approaches (RR 1.42, 95% CI 1.16–1.74; *I* ^2^ 94.8%, 95% CI 90.5%–97.1%; Figure 9) [29],[121],[125],[127].

Two studies reported the CD4 counts of participants found to be HIV-positive in a key population. Using standard lab diagnostics these studies reported a median CD4 count of 550 cells/µl among MSM [115] and 385 cells/µl among MSM, PWID, and FSW [105].

### Linkage to Care

**Figure 15:**
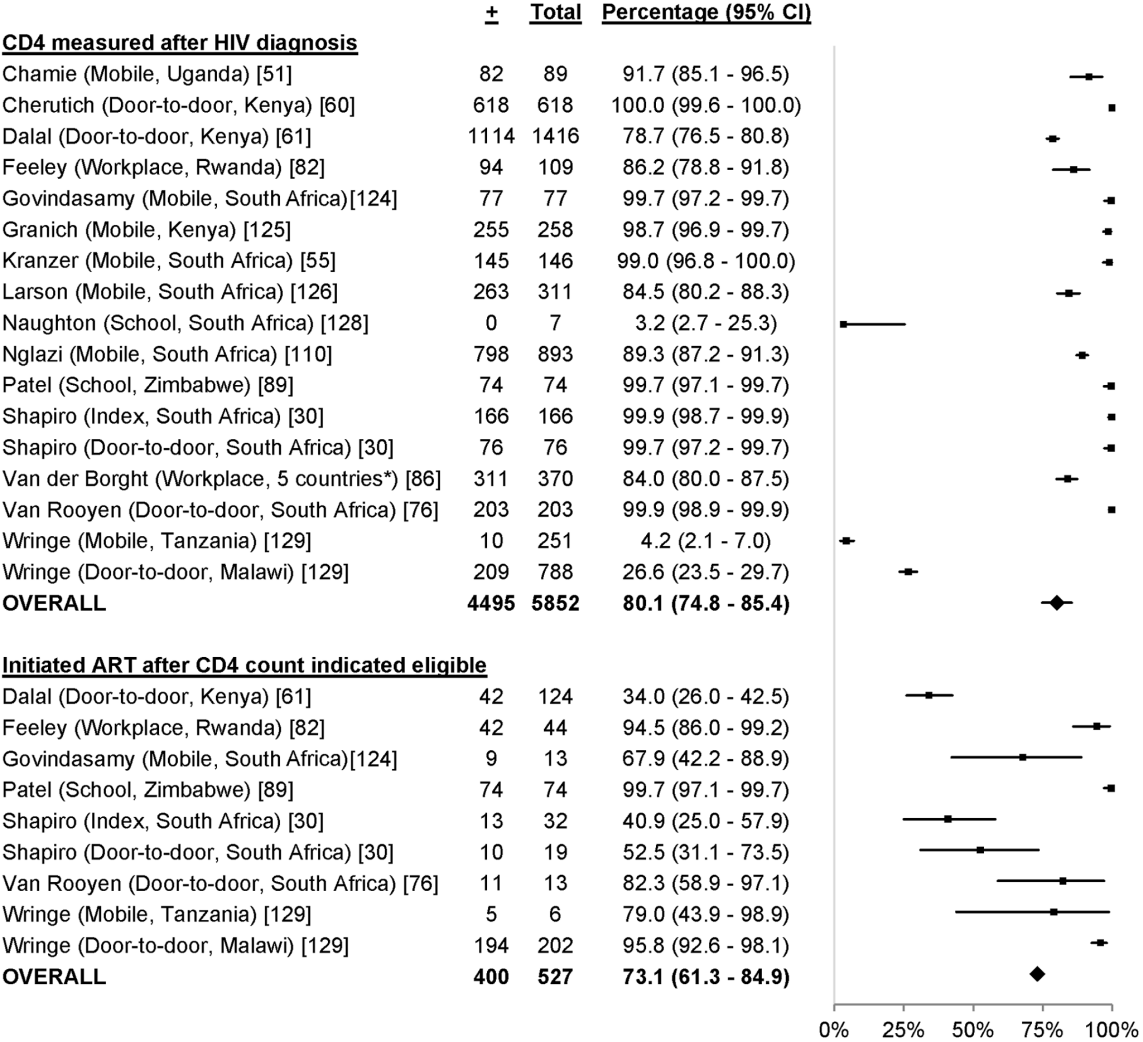
Linkage to care with community-based approaches to HTC. Asterisk: study included 14 workplace sites in the Democratic Republic of Congo, Rwanda, Burundi, Congo, and Nigeria.

17 studies, including 5,852 participants with HIV, reported linkage to care from HIV diagnosis to CD4 measurement [30],[51],[55],[60],[61],[76],[82],[86],[89],[110],[124]–[126],[128],[129]. Overall, 80.1% of participants had their CD4 count measured after HIV diagnosis (95% CI 74.8%–85.4%; *I* ^2^ 99.5%, 95% CI 99.4%–99.5%; Figure 15). Nine studies with 527 participants reported linkage to care from being eligible to ART to initiating ART [30],[61],[76],[82],[89],[124],[129]. Overall, 73.1% of participants initiated ART after their CD4 count indicated that they were eligible (95% CI 61.3%–84.9%; *I* ^2^ 96.9%, 95% CI 95.6%–97.9%; Figure 15).

Two studies, including 52 participants with HIV, reported linkage to care from HIV diagnosis to CD4 measurement in key populations. 12 of 15 MSM had their CD4 count measured after HIV diagnosis [115]. 26 of 37 MSM and/or PWID had their CD4 count measured after HIV diagnosis [105]. No studies reported linkage to care from being eligible for ART to initiating ART in key populations.

### Coverage

14 studies summarised HTC coverage among all people living in the testing site's catchment area [23],[51],[56],[63],[70],[71],[74],[81]. Coverage of HTC ranged from 5% to 93% depending on the type of approach used (Table 5). Mobile HTC available as part of multi-disease health campaigns achieved high coverage in the shortest period of time. Community-based HTC increased coverage of HTC relative to facility-based approaches (RR 7.07, 95% CI 3.52–14.22; *I* ^2^ 99.7%, 95% CI 99.7%–99.8%; Figure 9).

**Table 5:** Community coverage of voluntary HTC.

Study (Testing Approach)	Duration (Months)	Country	Year	Number Tested	Number Eligible	Percent Coverage
Sweat (mobile) [23]	42	Thailand	2007	10,464	11,290	93%
Lugada (mobile) [56]	0.23	Kenya	2008	47,173	51,178	92%
Chamie (mobile) [51]	0.16	Uganda	2007	4,343	6,300	69%
Wolff (door-to-door) [81]	1	Uganda	2001	1,078	1,591	68%
Naik (door-to-door) [70]	16	South Africa	2010	5,086	7,614	67%
Kimaiyo (door-to-door) [63]	7	Kenya	2009	90,062	143,284	63%
Negin (door-to-door) [71]	—	Kenya	2008	1,984	3,180	62%
Sweat (mobile) [23]	42	Zimbabwe	2007	6,579	10,700	61%
Tumwesigye (door-to-door) [74]	30	Uganda	2007	264,966	—	52%
Sweat (mobile) [23]	37	Tanzania	2007	2,832	6,250	45%
Sweat (facility-based) [23]	42	Thailand	2007	2,721	10,033	27%
Sweat (facility-based) [23]	37	Tanzania	2007	685	6,733	10%
Sweat (facility-based) [23]	42	Zimbabwe	2007	610	12,150	5%
Wolff (facility-based) [81]	12	Uganda	2000	79	1,591	5%

—, data not reported.

### HIV Incidence

One study reported HIV incidence [130]. There was a decreased risk of HIV infection in communities randomised to community-based testing relative to communities randomised to facility-based testing, although this estimate lacked statistical significance (RR 0.86, 95% CI 0.73–1.02; Figure 9).

### Cost per Person Tested

The cost per person tested ranged from US$2.45 to US$881.63 using different community-based testing approaches (Table 6) [29],[45],[71],[74],[107],[117],[122],[131]–[136]. The cost per person tested was US$2.45 to US$14.37 for door-to-door testing, US$3.26 to US$33.54 for mobile testing, US$12.91 for hospital testing, US$15.30 to US$203.04 for index testing, US$21.28 to US$29.56 for testing at a fixed HTC site, US$126.48 for church-based testing, US$92.83 to US$881.63 for community-based testing for key populations, and US$93.73 for testing at an HIV clinic. Due to the heterogeneity in health systems and HIV prevalence within and between countries, the cost per person identified with HIV was not included.

**Table 6:** Cost per person tested using different community-based testing approaches.

Study (Testing Approach)	Country	Components Included	Year	Number Tested	Total Costs (US Dollars)	Cost per Person Tested (US Dollars)	Cost per Person Tested (2012 US Dollars)
Molesworth (door-to-door) [68]	Malawi	Testing supplies	2007	11,172	$26,019	$2.33	$2.45
Edgil (mobile) [136]	Swaziland	Testing supplies	2011	152,000	$486,834	$3.20	$3.26
Tumwesigye (door-to-door) [74]	Uganda	Testing supplies, personnel, and transportation	2007	52,342	$367,792	$7.03	$7.77
Chamie (mobile) [51]	Uganda	Testing supplies, personnel, and buildings	2012	—	—	$8.27	$8.27
Menzies (door-to-door) [29]	Uganda	Testing supplies, personnel, transportation, vehicles, buildings, utilities, training, and equipment	2007	—	—	$8.29	$9.16
Negin (door-to-door) [71]	Kenya	Testing supplies, personnel, and transportation	2008	1,984	$17,569	$8.86	$9.43
Kahn (mobile)^a^ [131]	Kenya	Testing supplies, personnel, training, and contingency expenses	2008	—	—	$9.91	$10.55
Helleringer (door-to-door) [25]	Malawi	Testing supplies, personnel, transportation, buildings, utilities, and training	2007	1,183	$15,181	$12.83	$14.37
Menzies (hospital) [29]	Uganda	Testing supplies, personnel, transportation, vehicles, buildings, utilities, training, and equipment	2007	—	—	$11.68	$12.91
Menzies (index) [29]	Uganda	Testing supplies, personnel, transportation, vehicles, buildings, utilities, training, and equipment	2007	—	—	$13.85	$15.30
Grabbe (mobile) [107]	Kenya	Testing supplies, personnel, vehicles, buildings, utilities, and equipment	2007	—	—	$14.91	$16.47
Menzies (fixed HTC site) [29]	Uganda	Testing supplies, personnel, transportation, vehicles, buildings, utilities, training, and equipment	2007	—	—	$19.26	$21.28
Grabbe (fixed HTC site) [107]	Kenya	Testing supplies, personnel, vehicles, buildings, utilities, and equipment	2007	—	—	$26.75	$29.56
Terris-Prestholt (mobile) [135]	Uganda	Testing supplies, personnel, vehicles, buildings, and equipment	2001	4,425	$114,761	$25.93	$33.54
McConnel (church) [132]	South Africa	Testing supplies, personnel, utilities, training, buildings, office equipment, and publicity materials	2003	662	$67,248	$101.58	$126.48
Keenan (mobile for MSM, PWID, and FSW) [117]	US	Testing supplies, personnel, and transportation	2001	735	$52,744	$71.76	$92.83
Shrestha (HIV clinic) [122]	US	Testing and office supplies, personnel, transportation, utilities, building, vehicles, and recruitment costs	2005	855	$68,318	$79.90	$93.73
Shrestha (mobile for MSM, PWID, and FSW) [122]	US	Testing and office supplies, personnel, transportation, utilities, building, vehicles, and recruitment costs	2005	1,679	$276,218	$164.51	$192.98
Wykoff (index) [45]	US	Testing supplies, personnel, and transportation	1988	62	$6,500	$104.84	$203.04
Shrestha (mobile for transgender individuals and PWID) [134]	US	Testing and office supplies, personnel, transportation, building, utilities, and incentives	2007	301	$190,202	$631.90	$698.22
Shrestha (mobile for MSM and PWID) [133]	US	Testing and office supplies, personnel, transportation, and incentives	2007	817	$651,873	$797.89	$881.63

a Cost included CD4 measurement and 60 condoms.

—, data not reported.

### Potential Harms

No studies reported harm arising as a result of having been tested. 18 studies gave a description of the testers' experiences or listed reasons for tester refusal [47]–[49],[52],[58],[64],[71],[81],[82],[91],[102],[109],[113],[115],[137]–[139]. The studies discussed both the clients' positive testing experiences and their fears. Eight studies (including one targeting key populations) reported instances where participants refused HTC because of fear of status disclosure or stigma [52],[58],[64],[71],[81],[82],[102],[109]. In contrast, 12 studies (including three studies targeting key populations) specifically reported either no evidence of harm [47]–[49],[55],[91],[113],[115] or benefit through improved privacy or reduced stigma and fear [52],[82],[137]–[139].

### Quality Assessment

There was concern of selection bias in nine of the studies included in pooled analyses [22],[23],[62],[111],[119],[125], concern of confounding in five studies [27],[62],[108],[111],[119], and concern of measurement bias in five studies [81],[107],[111],[121],[127] (Table S2). The randomised trials appeared to have limited selection, attrition, and reporting bias; however, their lack of blinding made them susceptible to performance and detection bias [22],[23] (Table S3).

### Sensitivity Analyses

While there was high uptake for community-based approaches in most studies, there were several outliers with low uptake. To gauge whether these outliers influenced pooled uptake estimates and increased heterogeneity we conducted sensitivity analyses without them (Table 7). Although the pooled estimates increased and the CIs tightened without the outliers, there was still high heterogeneity using the *I* ^2^ statistic. There was potential for selection bias, confounding, and measurement bias in several of the observational studies identified (Table S2). To determine whether these studies introduced bias into our results we ran sensitivity analyses without them and found the results to be similar (Table 8).

**Table 7:** Pooled relative risks of community- versus facility-based HTC sensitivity analyses.

Outcome	Pooled RR (95% CI)	*I^2^* Statistic	Observational Studies Removed	Revised Pooled Estimate (95% CI)	Revised *I^2^* Statistic
Uptake	10.65 (6.27–18.08)	96.1%	[43]	13.99 (11.75–16.68)	N/A
Proportion of first-time testers	1.23 (1.06–1.42)	99.8%	[27],[29],[107],[108],[111]	1.12 (0.91–1.38)	99.9%
HIV positivity rate	0.59 (0.37–0.96)	99.6%	[27],[29],[43],[62],[87],[108],[119]–[121]	0.47 (0.22–1.02)	99.6%
Coverage	7.07 (3.52–14.22)	99.7%	[81]	5.71 (2.63–12.40)	99.8%

N/A, not applicable.

**Table 8:** Pooled uptake proportion sensitivity analyses.

HTC Approach	Pooled Estimate (95% CI)	*I^2^* Statistic	Outliers Removed	Revised Pooled Estimate (95% CI)	Revised *I^2^* Statistic
Index	88.2 (80.5–95.9)	99.7%	[30]	93.5 (89.1–97.9)	99.0%
Mobile	86.8 (85.6–88.1)	99.9%	[26],[52],[53]	97.9 (97.6–98.3)	98.5%
Door-to-door	80.0 (76.9–83.1)	99.9%	[30],[68],[77],[79],[81]	84.2 (81.8–86.6)	99.9%
Workplace	67.4 (32.8–100.0)	100%	[86]	76.9 (61.8–92.0)	99.8%
School	62.1 (39.6–84.5)	99.0%	[87]	71.9 (46.4–97.3)	99.4%

Outliers were defined as study estimates more than one standard deviation away from the pooled estimate.

## Discussion

This systematic review found that community-based HTC approaches were successful in reaching populations early in the course of HIV infection. The studies with facility-based comparator arms further suggest that community-based HTC reached populations earlier in the course of HIV infection than facility-based HTC. Earlier HIV diagnosis supports timely access to ART, which could improve life expectancy and reduce HIV transmission 140–142. Earlier HIV diagnosis linked to ART may also have important socioeconomic effects at the population level, including (1) reducing the number of orphans [143], (2) improving education and employment outcomes [144],[145], and (3) increasing the size of workforces [146],[147].

The HIV positivity rate among participants in community-based HTC approaches was generally lower than that among participants in facility-based HTC. This could be because (1) symptomatic people with HIV are more likely to visit health facilities, (2) healthcare workers are more likely to offer HTC to patients with symptoms that might be associated with HIV, and (3) the positivity rate of participants in community-based HTC is more likely to be representative of the general population. While obtaining a lower positivity rate may immediately be associated with increased numbers needing to be tested to identify people with HIV, community-based HTC increased the number of newly diagnosed people with HIV 4-fold in a randomised controlled trial, has the potential to decrease HIV stigma by normalising HIV testing, and is an opportunity to provide prevention interventions for HIV and other diseases to asymptomatic populations [23],[52],[82],[137]–[139]. The HIV positivity rate among key populations utilising community-based testing varied relative to the HIV positivity rate among key populations utilising facility-based HTC and requires further examination within different epidemiological contexts. Although few comparative cost data exist on the various HTC approaches, the reported estimates indicate that several community-based testing approaches are cheaper or similarly priced compared to facility-based HTC (Table 6).

Because many settings lack universal health coverage, other disease control strategies—such as the guinea worm eradication campaign [148], eradication campaigns against polio and measles [149],[150], and efforts to eliminate preventable blindness [151]—are built upon community-based elements for broader reach. Since community outreach efforts for these disease control strategies have largely been vertical in nature, some have suggested leveraging community-based HTC as a conduit for delivering other public health activities based on national burden of disease [152]. Multi-disease approaches may include the provision of vaccines, water filters, and malaria bed nets and screening for cardiovascular disease, diabetes, and pulmonary disease [153]. Settings implementing recent WHO guidance on community-based screening for tuberculosis and malaria could also consider multi-disease frameworks to improve efficiency [154],[155]. Including other public health activities based on national epidemiology, such as family planning and viral hepatitis screening and treatment, may also be appropriate. Indeed, 40 of the 117 studies meeting this review's eligibility criteria (34%) had a multi-disease component. Broadening community-based HTC to include preventive interventions and screening for other diseases could further improve cost-effectiveness [156].

In the studies reviewed, HTC uptake exceeded 80% in the mobile, index, self, and door-to-door testing approaches. While workplace and school-based testing could be an important approach in some settings, the uptake of these approaches was lower than that of other community-based approaches. Further research may improve their acceptability and could evaluate their impact on employment and education outcomes. Although there was no evidence of any harm resulting from being tested in community-based HTC approaches, there were reports of fear of status disclosure or stigma. Moreover, a recent report highlights the possibility of false positive diagnoses in settings (1) lacking a confirmation HIV test, (2) with poor training and supervision of community health workers, and (3) with insufficient quality control procedures [157]. These findings highlight the continuing need to adhere to validated testing algorithms and to address legal and human rights issues, and for the 5 Cs of good testing practices—informed consent, confidentiality, counselling, correct test results, and connection to prevention and care—to always be present [10].

There was variable uptake for community-based testing among key populations. The heterogeneity between studies likely relates to differences in the way HTC was offered. For example, the studies with the lowest uptake among key populations offered HTC only in combination with extensive behavioural surveys [92],[95]. The findings from this small number of studies cannot be generalised widely. Moreover, there were limited CD4 count and ART linkage data from these studies, indicating that caution may be needed when providing HTC to key populations in settings where they remain marginalised and stigmatised and where there are inadequate linkages to prevention and care services. It is also important to safeguard confidentiality and prevent possible coercion, discrimination, and other adverse consequences for key populations being offered HTC in community settings. Further operational research on community-based testing for key populations, including mobile peer-based models [120],[133],[158],[159], within this human rights framework is needed.

One of the benefits of community-based testing, especially door-to-door testing, is allowing couples and families to be counselled about their HIV status, behaviour change, ART, and prevention interventions together [160],[161]. Review of population-level HTC efforts suggest that implementation of community-based HTC could increase the number of couples receiving testing (Table 2). There were relatively limited data on HTC approaches for infants, children and adolescents. The door-to-door, mobile, school, and index community-based approaches have promise for these young populations, but further research could improve their operationalisation [43],. In addition to implementing provider-initiated HTC in all health facilities in generalised epidemics, introducing HIV testing at scheduled immunisation visits may facilitate earlier diagnosis linked to care [162].

Offering community-based HTC in addition to facility-based HTC increased knowledge of HIV status approximately 7-fold at the population level. Providing near universal knowledge of HIV status linked to prevention and care may impact HIV transmission networks through increased coverage of ART, increased male circumcision prevalence, increased utilisation of needle exchange programmes, increased utilisation of condoms, increased utilisation of pre-exposure prophylaxis, behavioural change, and increased coverage of opiate substitution therapy. A cluster-randomised trial detected a statistically non-significant 14% reduction in population incidence in communities where community-based HTC was available [130]. Since community-based HTC wasn't directly linked to prevention and care services in this trial, achieving and maintaining high levels of HTC coverage and maximising linkage to ART and other components of combination prevention could lead to more substantial reductions in population incidence [163]–[165].

Incidence reductions depend on high coverage of repeat testing among people at risk of HIV infection. WHO recommends that HIV-negative individuals with ongoing sexual behaviour and/or who inject drugs with partners of positive or unknown HIV status should be tested at least annually [166]. A high percentage of people reported being first-time testers with community-based approaches, and overall there was a higher proportion of first-time testers in community-based approaches than in facility-based approaches. In effective HTC programmes, the proportion of people reporting receiving their first test should decrease over time as a result of implementing WHO repeat testing recommendations [25]. Several studies assessed uptake in the context of repeat testing. In several generalised epidemic settings, uptake remained high among the general population [24],[25]. Conversely, uptake decreased among the general population in a concentrated epidemic, suggesting that HTC may need to be targeted to key populations on an ongoing basis in these settings [26].

This review found that 80% of participants in the community-based HTC studies where CD4 measurement was offered had their CD4 count measured after HIV diagnosis. CD4 measurement was facilitated by (1) point-of-care CD4 diagnostics, (2) collection of blood samples at the time of diagnosis, and (3) workplace programmes that had regular contact with participants because of their work schedules. This percentage was similar to the percentages reported in two systematic reviews evaluating CD4 measurement from facility-based testing (59%–72%) [167],[168], and supports the notion that high uptake of CD4 measurement can be achieved outside of health facilities when it is offered in combination with testing results. This review also found that 73% of participants initiated ART after their CD4 counts indicated that they were eligible. This proportion was comparable to previous estimates from two systematic reviews evaluating ART initiation rates from healthcare facilities (62%–68%) [167],[168]. Linkage from community-based HTC approaches to community-based treatment programmes could improve ART access and uptake and merits further exploration [169]–[171]. The data on linkage to prevention services, including linking men with negative results to male circumcision [57], were very limited. These linkages will be required to maximise the population benefits of community-based testing. Additional data on linkage to prevention services are urgently needed. Because self-testing achieves anonymous knowledge of status, no studies have been able to provide data on rates of linkage to care or prevention for people using this testing approach [172]. Nonetheless, self-testing may provide programmatic advantages in some settings and requires further research [173].

There are some methodological limitations that need to be considered when evaluating the impact of community-based HTC. One of the outcomes, first-time tester proportion, has potential for recall bias since it relies on participants to recall their history of HIV testing. Since all of the studies that included a facility-based HTC comparator arm did not indicate whether HTC was provider- or client-initiated, comparisons were made to facility-based HTC approaches irrespective of who initiated the interaction. Therefore, this review may not provide conclusive evidence of community-based HTC relative to provider-initiated HTC. While 73% of participants initiated ART after their CD4 count indicated they were eligible, all of the studies providing these data did not provide information on the timing of this outcome. Understanding how soon after diagnosis participants were able to initiate ART could help establish the efficiency of linkage systems. While this review summarises information from different community-based testing approaches globally, only six of the 117 studies identified were from Asia, indicating a need to expand community-based HTC research efforts in this region. Finally, given the complexity and expense of conducting cluster-randomised controlled trials, most of the studies meeting the eligibility criteria were observational. Although our analyses included data from randomised controlled trials, the potential for unmeasured confounding in observational studies makes attempts to establish causal effect more difficult.

The meta-analyses may have limitations in the statistical methodology used. Using the *I* ^2^ statistic, there was high heterogeneity for most meta-analyses. All analyses should be interpreted with respect to local epidemiology, social and cultural context, and the health systems organisation of the studies contributing data. Publication bias was not formally assessed, as analytical methods to test for publication bias, such as funnel plots and funnel plot asymmetry tests, may not be appropriate for observational data [174]. Multiple study estimates and standardised variable definitions are required to explore the contributors of heterogeneity for pooled estimates. Given that the same variables were not collected systematically in all studies, this assessment was not undertaken for this review.

In conclusion, many community-based approaches achieved high uptake of HTC. Costs and linkage to care appeared similar to those of facility-based HTC approaches. The lower yield of people with HIV relative to facility-based HTC approaches appears to be offset by increasing knowledge of status at the population level, which, combined with timely linkage to treatment and prevention services, could have population effects on life expectancy and HIV transmission. As countries develop their new national strategic plans and investment cases based on WHO and Joint United Nations Programme on HIV/AIDS strategic guidance [175], consideration should be given to increasing the proportion of people with HIV who know their status, with linkages to prevention and care, by offering community-based testing in addition to facility-based testing [176].

## Supporting Information

Protocol S1 **Systematic review protocol.**
(PDF)

Table S1 **Search strategy for all databases.**
(PDF)

Table S2 **Newcastle-Ottawa Quality Assessment Scale.** *Given that the distribution of possible confounders in randomised controlled trials is related to chance alone, randomised controlled trials were not assessed for confounding.
(PDF)

Table S3 **Bias assessment for randomised controlled trials.**
(PDF)

Table S4 **Characteristics of studies meeting inclusion criteria.** CE model, cost-effectiveness model; N/A, not applicable (e.g., gender data was not calculated for community-based testing only for MSM or FSW); N/R, not reported; OS, observational study; RCT, randomised controlled trial; STD, sexually transmitted disease; TB, tuberculosis; VCT, voluntary counselling and testing.
(PDF)

Text S1 **PRISMA checklist.**
(DOC)

## Abbreviations

ART: antiretroviral therapy: CI, confidence interval
FSW: female sex workers
HTC: HIV testing and counselling
MSM: men who have sex with men
PWID: people who inject drugs
RR: relative risk
WHO: World Health Organization
