# Supplementary material for: Towards Universal Voluntary HIV Testing and Counselling: A Systematic Review and Meta-Analysis of Community-Based Approaches
Source: PLoS Med. 2013 Aug 13;10(8):e1001496. doi: 10.1371/journal.pmed.1001496 (PMC3742447; doi:10.1371/journal.pmed.1001496)
Supplement: Protocol S1 — Systematic review protocol. (PDF) [file pmed.1001496.s001.pdf]

Towards universal voluntary HIV testing and counselling: a systematic review of community-based approaches

Introduction

HIV is a leading cause of death and hinders economic development globally [1]. In 2010 there were 2.7 million new infections and 1.8 million deaths among the 34 million people with HIV worldwide [2]. Antiretroviral therapy (ART) prevents HIV-related mortality and has transformed HIV infection into a manageable chronic condition [3]. Moreover, ART can virtually eliminate vertical and heterosexual HIV transmission [4, 5]. To harness ART's population effects on life expectancy and transmission, people must be diagnosed and enrolled into care early in the course of HIV infection [3]. Learning HIV status can also decrease unprotected sex and provide an opportunity for males without HIV to consider circumcision to reduce susceptibility to HIV [6, 7].

Since 2007 WHO has recommended provider-initiated HIV testing and counselling (HTC) to all persons seen in all health facilities in generalised (i.e. antenatal HIV prevalence ≥ 1%) epidemics and in specific services in concentrated epidemics [8]. WHO also recommends couples testing and counselling with support for mutual disclosure in all health facilities and in other settings [9]. Since rapid HIV tests are highly sensitive and specific, they should be used where feasible [10].

Within the last decade, HTC has significantly expanded in low and middle income countries through both provider-initiated and client-initiated HTC approaches. In 2010, 35% of pregnant women were tested for HIV and overall more than 79 million HIV tests were performed [11]. However, the latest Demographic and Health Surveys indicate that only 11% of people aged 15-49 years in generalised epidemics received an HIV test in the previous year [12]. Late diagnosis and poor linkages to care largely contribute to most people with HIV accessing ART late in the course of their disease, which leads to avoidable morbidity, mortality, and HIV transmission [13].

The reasons for limited uptake of HTC are complex and include service-level barriers (shortages of trained health workers, restricted and limited availability of free tests, frequent stock-outs of tests, and a limited number of testing sites) and patient-level barriers (long distances to clinics, limited funds for transportation, lack of HIV awareness, and fear of a positive result due to enduring stigma) [14]. Access to and uptake of HTC also varies by gender and population group. For example, in generalised epidemics women have higher rates of testing than men, largely because of their more frequent contact with health services. Moreover, adolescents are also poorly serviced by many programmes which primarily rely on facility-based HTC. Although provider-initiated HTC in antenatal care clinics has often been successfully implemented in

concentrated epidemics, access and uptake of HTC by key and vulnerable populations remains poor with current implementation approaches. This has highlighted the need to consider community-based HTC approaches to support increased equitable access and earlier diagnosis.

Over the past 20 years a number of countries have offered HTC outside of health facilities through stand-alone voluntary counselling and testing sites. However, this approach is often costly, favours those with higher income and literacy rates, does not reach all people at risk of HIV infection, and is sometimes associated with stigma [14]. Recently, other community-based approaches have been developed to support increased and potentially more equitable access to HIV testing at the district-level [15, 16]. Previous research has systematically reviewed the impact of house-to-house HTC in developing countries [17]; however, substantial evidence has accumulated since this review was published. Moreover, the evidence regarding the impact of other community-based HTC approaches has not undergone formal systematic review or synthesis. The objective of this study is to systematically review all community-based approaches of HIV testing and counselling.

Methods

Conduct of systematic review

This systematic review will be conducted in accordance with PRISMA guidelines [18]. The PubMed, Embase, African Index Medicus, Index Medicus for the Eastern Mediterranean Region, Index Medicus for the Southeast Asia Region, Western Pacific Region Index Medicus, and Latin American and Caribbean Health Science Literature databases will be systematically searched without language, publication, date, or any other limits. The WHO International Clinical Trials Registry Platform, the Cochrane Central Register of Controlled Trials, the International Standard Randomised Controlled Trial Number Register, and ClinicalTrials.gov will be searched for future and on-going studies. Experts in the field will be contacted to identify unpublished research and on-going studies.

Search strategy and selection criteria

The search strategies (Table 1) were designed with a librarian to identify studies including community-based HIV testing and counselling. Per recommendations from the PRISMA Group, eligibility criteria were based on key study characteristics: population, intervention, comparator, outcome, and design [18]. Specifically, studies were included when (1) the study population included people in generalised or concentrated HIV epidemics, (2) the intervention was

community and facility-based HTC, (3) the comparator was facility-based HTC alone, (4) the outcomes were CD4 value at diagnosis; testing uptake (i.e. the proportion of the study population accepting HTC); community HIV incidence; linkage to care rates; proportion of people receiving their first HIV test; or cost effectiveness, and (5) the study design was a randomised trial or observational cohort study. Community-based HTC approaches were defined as those outside of health facilities (Table 1). Given the difficulties and expense of conducting large-scale studies for community-based HTC, studies without a comparator arm will also be included. Three of the investigators, ABS, NF, and WA, will independently screen abstracts of all identified articles and then match the full texts of all articles selected during screening against the inclusion criteria. Articles failing to meet these inclusion criteria, or featuring fixed stand-alone voluntary testing and counselling sites, will be excluded from this review.

Data extraction

ABS will complete the data extraction using a standardised extraction form comprising five tables. The first table will summarise the characteristics of study participants. The second table will include information on the community-based testing approaches, including: pre-test demand creation, multi-disease components, study design, linkage to care, provision of incentives, and required number of visits. The third table will summarise the reported outcomes.

Subsequent tables will include studies with a comparator arm. The fourth table will summarise study methods (analytical model used and variables included in the model, information on the comparator arm, and information on outcome ascertainment). The final table will focus on quality assessment.

Statistical analyses

For statistical analyses, studies will be stratified based on the community-based testing approach. A funnel plot with the effect measures on the *x*-axis and standard error of the log for the effect measures on the *y*-axis will be created to assess publication bias and the Egger and Begg tests will be used to test the funnel plot's symmetry. If studies are similar enough to combine, meta-analyses will be performed and statistical heterogeneity will be assessed. Effect measures will be entered as the natural log of the effect measure and standard error as the natural log of (95% upper limit ÷ 95% lower limit) ÷ 3.92 [19]. Fixed-effect models assume that the magnitude and direction of an interventions' effects are identical across studies and that observed differences

among study results are due solely to chance [20]. Random-effects models assume that the magnitude and direction of an interventions' effects are not identical but follow a distribution [20]. Since it is possible that the magnitude and direction of community HTC's impact could differ for reasons other than chance, random-effects models will be used for all analyses. An I-squared statistic will be used to measure heterogeneity [21]. I-squared statistics near 25% indicate low heterogeneity, values near 50% indicate moderate heterogeneity, and those above 75% indicate high heterogeneity [22]. If there is moderate to significant heterogeneity in estimates, potential causes, including pre-test community sensitisation; study design; linkage to care; provision of incentives; and required number of visits, will be explored using sensitivity analyses. STATA version 10.0 will be used for all analyses.

Quality assessment

For the quality assessment, studies will be stratified based on study design (i.e. randomised controlled trial or observational study). Per recommendations from the Cochrane Collaboration, the Collaboration's 'Risk of bias' tool will be used to assess bias in randomised trials with a comparator arm [20]. This tool rates studies based on six criteria in four sources of bias. The presence of random sequence generation for allocation into intervention and comparator arms, and attempts to conceal this allocation, will be used to gauge selection bias. Blinding of study participants, personnel, and outcome assessment during the conduct and analysis of the studies will be used to gauge performance and detection bias. Incomplete outcome data, through review of participants excluded from outcome analyses or lost to follow-up, will be used to gauge attrition bias. Selective reporting of outcomes, time-points, subgroups, or analyses, will be used to gauge reporting bias. A criterion for other forms of bias will also be used. Based on these criteria, studies will be scored out of 100%.

Per recommendations from the Cochrane Collaboration [20], the *Newcastle-Ottawa Quality Assessment Scale* will be used to assess bias in observational studies with a comparator arm [23]. This scale rates studies based on eight criteria in three sources of bias. Each criterion is worth one point except confounding, which is worth two points. Selection bias will be assessed using four criteria: (1) representativeness of the cohort in the intervention arm the average person in the community from which study participants were drawn, (2) representativeness of the cohort in the comparator arm to the intervention arm, (3) ascertainment of HTC, and (4) demonstration that the outcome was not present at the start of follow up. Adjustment for a patient-level barrier (distance to testing site, income level, or education level) will be used to judge whether

appropriate methods were used to address confounding. Measurement bias will be assessed using three criteria: (1) assessment of outcome, (2) adequate follow-up to detect the outcome, and (3) ≤ 30% of participants lost to follow up during the study. Based on these criteria, studies will be scored out of 100%.

For randomised trials and observational cohort studies, studies scoring ≥ 67% will be considered to have a low risk of bias, those scoring 34-66% will be considered to have an unclear risk of bias, and those ≤ 33% will be considered to have a high risk of bias.

The quality of evidence will be assessed using the *Grades of Recommendation, Assessment, Development, and Evaluation* (GRADE) system to guide programme managers and other policy makers on national community-based HTC strategies (Appendix, [24]).

Table 1. Search strategy for all databases.

Search number	Search terms
1	HIV
2	human immunodeficiency virus
3	1 or 2
4	counsel*
5	test
6	testing
7	tested
8	5 or 6 or 7
9	community
10	home
11	house
12	door
13	mobile
14	campaign
15	bar
16	workplace
17	business
18	church
19	temple
20	active
21	school
22	highway
23	brothel
24	bathhouse
25	festival
26	outreach
27	van
28	bicycle
29	9 or 10 or 11 or 12 or 13 or 14 or 15 or 16 or 17 or 18 or 19 or 20 or 21 or 22 or 23 or 24 or 25 or 26 or 27 or 28
30	3 and 4 and 8 and 29

REFERENCES

1. Lopez AD, Mathers CD, Ezzati M, Jamison DT, Murray CJL. Global Burden of Disease and Risk Factors. New York, NY: The World Bank and Oxford University Press; 2006.
2. UNAIDS. 2011 World AIDS Day report. 2011 [cited 23 November 2011]; Available from: http://www.unaids.org/en/media/unaids/contentassets/documents/unaidspublication/2011/JC2216_WorldAIDSday_report_2011_en.pdf
3. Mills EJ, Bakanda C, Birungi J, Chan K, Ford N, et al. Life expectancy of persons receiving combination antiretroviral therapy in low-income countries: a cohort analysis from Uganda. Ann Intern Med. 2011; **155**(4): 209-16.
4. Cohen MS, Chen YQ, McCauley M, Gamble T, Hosseinipour MC, et al. Prevention of HIV-1 Infection with Early Antiretroviral Therapy. N Engl J Med. 2011; **365**(6): 493-505.
5. Shapiro RL, Hughes MD, Ogwu A, Kitch D, Lockman S, et al. Antiretroviral regimens in pregnancy and breast-feeding in Botswana. N Engl J Med. 2010; **362**(24): 2282-94.
6. Mills E, Cooper C, Anema A, Guyatt G. Male circumcision for the prevention of heterosexually acquired HIV infection: a meta-analysis of randomized trials involving 11,050 men. HIV Med. 2008; **9**(6): 332-5.
7. Denison JA, O'Reilly KR, Schmid GP, Kennedy CE, Sweat MD. HIV voluntary counseling and testing and behavioral risk reduction in developing countries: a meta-analysis, 1990--2005. AIDS Behav. 2008; **12**(3): 363-73.
8. WHO. Guidance on provider-initiated HIV testing and counselling in health facilities. 2007 [cited 2011 7 February]; Available from: http://whqlibdoc.who.int/publications/2007/9789241595568_eng.pdf
9. WHO. Couples HIV Testing and Counselling including Antiretroviral Therapy for Treatment and Prevention in Serodiscordant Couples. 2012 [cited 21 May 2012]; Available from: http://whqlibdoc.who.int/publications/2012/9789241501972_eng.pdf
10. Branson BM. State of the art for diagnosis of HIV infection. Clin Infect Dis. 2007; **45 Suppl 4**: S221-5.
11. WHO. Global HIV/AIDS Response: Epidemic update and health sector progress towards universal access. 2011 [cited 15 December 2011]; Available from: http://whqlibdoc.who.int/publications/2011/9789241502986_eng.pdf
12. WHO. Towards universal access: Scaling up priority HIV/AIDS interventions in the health sector. 2010 [cited 11 April 2011]; Available from: http://whqlibdoc.who.int/publications/2010/9789241500395_eng.pdf
13. Mugglin C, Althoff K, Wools-Kaloustian K, Sterne J, Nash D, et al. Immunodeficiency at the start of ART: Global View. 19th Conference on Retroviruses and Opportunistic Infections; 2012 5-8 March 2012; Seattle, United States of America; 2012.
14. Matovu JK, Makumbi FE. Expanding access to voluntary HIV counselling and testing in sub-Saharan Africa: alternative approaches for improving uptake, 2001-2007. Trop Med Int Health. 2007; **12**(11): 1315-22.
15. Lugada E, Millar D, Haskew J, Grabowsky M, Garg N, et al. Rapid implementation of an integrated large-scale HIV counseling and testing, malaria, and diarrhea prevention campaign in rural Kenya. PLoS One. 2010.
16. Tumwesigye E, Wana G, Kasasa S, Muganzi E, Nuwaha F. High uptake of home-based, district-wide, HIV counseling and testing in Uganda. AIDS Patient Care STDS. 2010; **24**(11): 735-41.
17. Bateganya MH, Abdulwadud OA, Kiene SM. Home-based HIV voluntary counseling and testing in developing countries. Cochrane Database Syst Rev. 2007; (4): CD006493.
18. Liberati A, Altman DG, Tetzlaff J, Mulrow C, Gotzsche PC, et al. The PRISMA statement for reporting systematic reviews and meta-analyses of studies that evaluate health care interventions: explanation and elaboration. PLoS Med. 2009; **6**(7): e1000100.

19. Cooper H, Hedges L. The Handbook of Research Synthesis. New York, NY: Russell Sage Foundation; 1994.
20. The Cochrane Collaboration. Cochrane Handbook for Systematic Reviews of Interventions. [cited 19 April 2011]; Available from: http://www.cochrane-handbook.org/
21. Higgins JP, Thompson SG. Quantifying heterogeneity in a meta-analysis. Stat Med. 2002; **21**(11): 1539-58.
22. Higgins JP, Thompson SG, Deeks JJ, Altman DG. Measuring inconsistency in meta-analyses. Bmj. 2003; **327**(7414): 557-60.
23. Wells G, Shea B, O'Connell D, Peterson J, Welch V, et al. The Newcastle-Ottawa Scale (NOS) for assessing the quality of nonrandomised studies in meta-analyses. [cited 23 February 2011]; Available from: http://www.ohri.ca/programs/clinical_epidemiology/oxford.asp
24. Schünemann H, Brozek J, Oxman A, editors. GRADE Handbook: for grading the quality of evidence and the strength of recommendations. 2009 [cited 29 March 2012]; Available from: www.who.int/hiv/topics/mtct/grade_handbook.pdf

APPENDIX

For systematic reviews, the GRADE approach defines the quality of a body of evidence as the extent to which one can be confident that an estimate of effect or association is close to the quantity of specific interest. The quality rating across studies has four levels: high, moderate, low, or very low. High quality indicates that further research is very unlikely to change our confidence in the estimate of effect. Moderate quality indicates that further research is likely to have an important impact on our confidence in the estimate of effect and may change the estimate. Low quality indicates that further research is very likely to have an important impact on our confidence in the estimate of effect and is likely to change the estimate. Very low quality indicates that any estimate of effect is very uncertain. By default randomised trials are categorised as high quality and can be downgraded while cohort studies are categorised as low quality and can be upgraded or downgraded. The GRADE Profiler software will be used for performing the GRADE assessment (GRADEprofiler version 3.2.2).

There are five factors that can decrease the quality of a body of evidence. The first factor is major limitations in study design or execution that are likely to result in a biased assessment of the effect estimate. This factor will be gauged by assessing the risk of bias across studies. When the proportion of information from studies at high risk of bias is sufficient to affect the interpretation of results, the risk of bias across studies will be 'high'. When most data included in the GRADE review is insufficient to affect the interpretation of results, the risk of bias across studies will be 'low'. When most information included in the GRADE review is from studies at an unclear risk of bias, the risk of bias across studies will be 'unclear'. 'Low' risk of bias will indicate 'no limitation', an 'unclear' risk of bias will indicate 'no limitation' or 'serious limitation', and a 'high' risk of bias will indicate 'serious limitation' or 'very serious limitation.' The second factor that can decrease the quality of a body of evidence is indirectness of evidence. Indirectness of evidence refers to bodies of literature that do not correspond to the population, intervention, comparator, and outcome specified in the inclusion criteria. The third factor that can decrease the quality of a body of evidence is inconsistency of study results. This would primarily be when studies yield widely different estimates of effect in terms of heterogeneity or variability in results. The fourth factor that can decrease the quality of a body of evidence is imprecision of results, i.e. when there are few participants, few events, and wide confidence intervals. The fifth and final factor that can decrease the quality of a body of evidence is high probability of publication bias. This would be when investigators fail to publish studies or outcomes on the basis of their results.

There are three factors that can increase the quality level of a body of evidence. The first factor is a large magnitude of effect. In the absence of plausible confounders, a large effect (i.e. $RR > 2$ or $RR < 0.5$) increases the quality one level while a very large effect (i.e. $RR > 5$ or $RR < 0.2$) increases the quality two levels. The second factor is plausible confounding that reduces the effect demonstrated in the included studies. The third factor is the presence of a dose-response gradient.
