# Supplementary material for: Towards Universal Voluntary HIV Testing and Counselling: A Systematic Review and Meta-Analysis of Community-Based Approaches
Source: PLoS Med. 2013 Aug 13;10(8):e1001496. doi: 10.1371/journal.pmed.1001496 (PMC3742447; doi:10.1371/journal.pmed.1001496)
Supplement: Table S1 — Search strategy for all databases. (PDF) [file pmed.1001496.s002.pdf]

Search number	Search terms
1	HIV
2	human immunodeficiency virus
3	1 or 2
4	counsel*
5	test
6	testing
7	tested
8	5 or 6 or 7
9	community
10	home
11	house
12	door
13	mobile
14	campaign
15	bar
16	workplace
17	business
18	church
19	temple
20	active
21	school
22	highway
23	brothel
24	bathhouse
25	festival
26	outreach
27	van
28	bicycle
29	9 or 10 or 11 or 12 or 13 or 14 or 15 or 16 or 17 or 18 or 19 or 20 or 21 or 22 or 23 or 24 or 25 or 26 or 27 or 28
30	3 and 4 and 8 and 29
