# Supplementary material for: Towards Universal Voluntary HIV Testing and Counselling: A Systematic Review and Meta-Analysis of Community-Based Approaches
Source: PLoS Med. 2013 Aug 13;10(8):e1001496. doi: 10.1371/journal.pmed.1001496 (PMC3742447; doi:10.1371/journal.pmed.1001496)
Supplement: Table S2 — Newcastle-Ottawa Quality Assessment Scale. *Given that the distribution of possible confounders in randomised controlled trials is related to chance alone, randomised controlled trials were not assessed for confounding. (PDF) [file pmed.1001496.s003.pdf]

	Selection bias			Confounding	Measurement bias		
Author (Testing strategy, country)	Representativeness of the cohort in the community-based HTC arm the average person in the community from which study participants were drawn	Representativeness of the cohort in the facility-based HTC arm to the cohort in the community-based HTC arm	HTC use documented	Community-based and facility-based HTC arms are comparable with respect to a patient-level barrier (distance to testing site, income level, or education level)*	Cases confirmed through two tests in countries with HIV prevalence > 5% and through three tests in countries with HIV prevalence < 5%	Adequate follow-up to learn HIV status (one visit for rapid tests and two visits for others)	Test results available for > 70% of those accepting HTC
Ahmed (Mobile, Nigeria) [27]	1	1	1	0	1	1	1
Coates (Mobile, South Africa, Tanzania, and Zimbabwe) [130]	1	1	1	NA	1	1	1
Corbett (Workplace, Zimbabwe) [22]	0	1	1	NA	1	1	0
Gonzalez (Door-to-door, Mozambique) [62]	1	0	1	0	1	1	1
Grabbe (Mobile, Kenya) [107]	0	1	1	2	1	1	0
Granich (Mobile, Kenya) [125]	0	0	1	2	1	1	1
Hood (Mobile, Botswana) [119]	1	0	1	0	1	1	1
Lahuerta (Mobile, Guatemala) [108]	1	0	1	0	1	1	1
Lugada (Index, Uganda) [43]	1	1	1	2	1	1	0
McCoy (Mobile, USA) [120]	0	1	1	NA	1	1	1
Menzies (Index, Uganda) [29]	1	1	1	2	1	1	1
Menzies (Door-to-door, Uganda) [29]	1	1	1	2	1	1	1
Spielberg (Mobile, USA) [111]	0	0	1	0	1	1	0
Sweat (Mobile, Tanzania) [23]	1	1	1	NA	1	1	1
Sweat (Mobile, Zimbabwe) [23]	1	1	1	NA	1	1	1
Sweat (Mobile, Thailand) [23]	0	1	1	NA	1	1	1
van Schaik (Mobile, South Africa) [121]	1	0	1	2	0	1	1
Wachira (Door-to-door, Kenya) [127]	0	1	1	2	0	0	0
Wolff (Door-to-door, Uganda) [81]	0	0	1	2	1	1	0
