# Supplementary material for: Towards Universal Voluntary HIV Testing and Counselling: A Systematic Review and Meta-Analysis of Community-Based Approaches
Source: PLoS Med. 2013 Aug 13;10(8):e1001496. doi: 10.1371/journal.pmed.1001496 (PMC3742447; doi:10.1371/journal.pmed.1001496)
Supplement: Table S3 — Bias assessment for randomised controlled trials. (PDF) [file pmed.1001496.s004.pdf]

Author (Testing strategy, country)	Adequate sequence generation (selection bias)	Allocation concealment (selection bias)	Blinding of participants, personnel, and outcome assessors (performance and detection bias)	Incomplete outcome data addressed (attrition bias)	Free of selective reporting (reporting bias)	Free of other bias
Corbett (Workplace, Zimbabwe) [22]	1	1	0	1	1	1
Coates and Sweat (Mobile, Tanzania, Zimbabwe, South Africa, and Thailand) [23, 130]	1	1	0	1	1	1
