# Supplementary material for: Towards Universal Voluntary HIV Testing and Counselling: A Systematic Review and Meta-Analysis of Community-Based Approaches
Source: PLoS Med. 2013 Aug 13;10(8):e1001496. doi: 10.1371/journal.pmed.1001496 (PMC3742447; doi:10.1371/journal.pmed.1001496)
Supplement: Table S4 — Characteristics of studies meeting inclusion criteria. CE model, cost-effectiveness model; N/A, not applicable (e.g., gender data was not calculated for community-based testing only for MSM or FSW); N/R, not reported; OS, observational study; RCT, randomised controlled trial; STD, sexually transmitted disease; TB, tuberculosis; VCT, voluntary counselling and testing. (PDF) [file pmed.1001496.s005.pdf]

Study	Publication Year	Year Study Conducted	Study Design (Location)	Region	Testing Approach Used (Key Population Targeted)	Study Description	Demand Creation	Incentives	Multi-Disease	Linkage to Care	Required Number of Visits to Obtain Results	Total Tested	Number Female	Percent Female Intervention (Percent Female Comparator)
Armbruster [41]	2011	2007–2008	OS (Likoma, Malawi)	Africa	Index	Inhabitants with HIV 18–49 y were asked to provide the names of up to five of their most recent sexual contacts. Sexual contacts were located, and HTC was offered.	None	None	None	None	1	127	N/R	N/R
Colindres [42]	2008	September 2005–February 2006	OS (Mbale, Mulago, and Mbarare, Uganda)	Africa	Index	A pamphlet was provided to family members of people with HIV providing details on home-based HTC. HTC was then offered to family members of people with HIV.	None	None	Insecticide-treated bed nets, supplies for household water treatment and safe storage, condoms, and cotrimoxazole for participants with HIV (total valued at US$19.14).	None	1	112	85	75.89%
Lugada [43]	2010	February 2005–February 2007	OS (Jinja, Kamuli, Iganga, Mayuge, and Mukono districts in southeastern Uganda)	Africa	Index	Household members of people with HIV were offered home-based testing or free vouchers for testing in the clinic. HIV testing was performed by trained lay field officers in the home arm and by laboratory technicians in the clinic arm.	None	None	None	ART indicated if HIV-positive family members.	1	4,798	2,685	55.96% (54.02%)
Menzies [29]	2009	June 2003–September 2005	OS, CE model (Uganda)	Africa	Index	Retrospective cohort study to get demographics and results of individuals receiving HTC from several HTC programmes: included index HTC as the intervention and hospital-based HTC as the comparator.	None	None	None	Hospital care if hospital-based HTC, referral if other types of HTC.	2	2,011	1,043	51.86% (62.36%)
Nelson [46]	2012	2009–2010	OS (Lima, Peru)	Central and South America	Index	Community health workers arranged HIV testing at the home (or alternative location) for people with TB and their household members.	None	None	None	None	1	98	41	40.60%
Shapiro [30]	2012	February–September 2009	OS (Matlosana municipality, North West Province, South Africa)	Africa	Index	Household members of people with TB infection (87% of whom were coinfected with HIV) were tested for HIV by a nurse and lay counsellors	None	None	Symptom screening (cough, fever, night sweats, and/or weight loss) and sputum assessment for TB.	Appointments and referrals to the ART clinic for participants with HIV and CD4 counts < 250 cells/μl.	1	2,843	1,644	57.83%
Were [80]	2006	May 2003 and December 2004	OS (Tororo and Busia, Uganda)	Africa	Index	Household and family members of people with HIV who were enrolled in home-based ART programmes were offered testing. Testing was also offered to family members under the age of 18 y.	None	None	None	Clinically eligible individuals received ART.	2	2,348	1,218	51.87%
Wykoff [45]	1988	1987	OS (South Carolina, US)	North America	Index	Starting with one index patient, people with HIV were asked to name all of their sexual and intravenous drug partners within the last 24 mo. This process was followed until no more contacts	None	None	None	HIV-positive individuals were referred to primary care physicians.	2	63	1	1.59%

						were found. Identified individuals were tested and counselled.								
Angotti 1 [24]	2009	2004	OS (three districts in Malawi)	Africa	Door-to-door	Randomly selected households in 120 villages were selected to participate. Door-to-door HTC was offered by trained counselors.	None	None	None	None	2	2,894	N/R	N/R
Angotti 2 [24]	2009	2006	OS (three districts in Malawi)	Africa	Door-to-door	Same as Angotti 1 [24].	Before testing was offered, counselors met with village leaders to organise meetings in which the forthcoming visits were explained and HTC was demonstrated.	None	None	None	1	2,748	N/R	N/R
Cherutich [60]	2012	2007	OS (Kenya)	Africa	Door-to-door	HIV testing data were obtained from the Kenya AIDS Indicator Survey. Participants were tested at home and asked about previous testing history.	None	None	Participants were asked about history of herpes simplex virus 2 and gonorrhea infection.	None	2	15,853	9,096	57.38%
Dalal [61]	2013	2008	OS (rural western Kenya)	Africa	Door-to-door	Door-to-door HIV testing and counselling was performed. Adolescents 13 y and older were included in the study, and children below 13 y were included if they had a biological mother who was known to be HIV-positive or deceased. Rapid testing was performed with finger stick.	Community mobilisers were identified and used to provide basic HIV testing information and create a mobilisation protocol.	None	None	Persons newly diagnosed were referred to care services.	1	19,966	N/R	56.38%
Gonzalez [62]	2012	N/R	OS (Mozambique)	Africa	Door-to-door	Participants were selected from an already in place national surveillance system, and they were offered rapid HIV testing and counselling in their homes.	None	None	None	None	1	722	366	50.69%
Helleringer 1 [25]	2012	February 2006	OS (Likoma, Malawi)	Africa	Door-to-door	The Likoma National Study investigates sexual networks and HIV transmission. The Likoma National Study includes a census, a survey, and home-based HTC. This article examined the results of the Likoma National Study in two campaigns.	Sensitisation meetings were conducted to explain the importance of HTC, demonstrate HTC procedures, provide information about access to ART, and answer questions.	None	None	Individuals with HIV-positive results were referred to the Likoma District Hospital for treatment and access to care services.	1	597	340	56.95%

Helleringer 2 [25]	2012	August 2007–April 2008	OS (Likoma, Malawi)	Africa	Door-to-door	Same as Helleringer 1 [25].	Same as Helleringer 1 [25].	None	None	Same as Helleringer 1 [25].	1	586	346	59.04%
Jurgensen [137]	2013	2009–2011	RCT (Monze district, Zambia)	Africa	Door-to-door	Adults were offered VCT in their homes by lay counsellors. Counsellors were trained for testing and counselling in the home setting. This article reported the effect of this testing approach on community-level stigma through pre- and post-test surveys.	Discussions and in-depth interviews with traditional leaders and community members. Information about door-to-door testing also broadcast on the radio.	None	None	None	1	N/R	N/R	N/R
Kimaiyo [63]	2010	2007–2009	OS (western Kenya)	Africa	Door-to-door	Counsellors visited homes and offered HTC to individuals ≥13 y. HTC was offered to children <13 y if the mother was HIV-positive or had an unknown HIV status. Counsellors educated all eligible individuals on HTC and continued with pre-test counselling if consent was obtained.	Campaigns were organised to create awareness and encourage community participation. Community mobilisation teams including a village elder, an adult, or a youth representative requested permission for counsellors to visit participants' homes.	None	None	Persons testing HIV-positive were referred for care.	1	138,026	73,837	53.49%
Kranzer [64]	2008	November 2005–August 2006	OS (Karonga, Malawi)	Africa	Door-to-door	Door-to-door HTC was offered to individuals between the ages of 18 and 59 y. Individual information was obtained from the participants through the use of interviews and physical examinations.	None	None	None	None	2	1,387	755	54.43% (53.79%)
Kyaddondo [138]	2012	2008–2009	OS (Kenya and Uganda)	Africa	Door-to-door	Door-to-door HTC was provided by 29 teams of providers, each with a counsellor, a laboratory technician, and mobilisers. HTC was provided free of charge, using rapid test kits, with immediately disclosed results.	Community mobilizers (i.e., local community leaders) informed individual households, public announcements at churches and	None	None	None	1	281	182	64.80%

							mosques, and talk shows on the local FM radio station.						
Maheswaran [28]	2012	February–June 2009	OS (KwaZulu-Natal, South Africa)	Africa	Door-to-door	For home testing, a team of six HIV counsellors visited every single household in the specified areas. All participants were provided with educational leaflets and condoms.	Before implementation, community mobilisation and education was achieved through a series of meetings with local political and traditional leaders and distribution of information leaflets and road shows to market the service.	None	None	Participants testing positive for HIV were provided with referral letters to the local HIV clinic for further follow-up.	1	1,585	1,095	69.09%
Matovu [65]	2002	April 1999–January 2000	OS (Rakai, Uganda)	Africa	Door-to-door	Door-to-door HTC was offered to adults between the ages of 15 and 49 y in 56 study communities in Uganda. In addition to this, participants were given an extensive questionnaire about their knowledge, attitudes, and behaviour related to HIV.	None	None	None	None	2	11,709	6,582	56.21%
Medley [66]	2013	2008–2009	OS (western Kenya)	Africa	Door-to-door	Adult residents (aged >13 y) in the Health and Demographic Surveillance System in Kisumu, Kenya, were offered HTC.	None	None	None	Referral letters with a list of all nearby health facilities offering HIV care and treatment services.	1	9,895	5,739	58.00%
Menzies [29]	2009	June 2003–September 2005	OS, CE model (Uganda)	Africa	Door-to-door	Retrospective cohort study to get demographics and results of individuals receiving HTC from one of several HTC programmes: door-to-door HTC is considered here as the intervention group, and testing at a stand-alone centre is considered as the comparator group.	None	None	None	Referral to care.	2	49,470	25,187	50.91% (48.74%)
Michelo [67]	2006	2003	OS (Kapiri Mposhi, Chelstone, and Lusaka, Zambia)	Africa	Door-to-door	Household members were counselled and offered HTC. Consent was obtained prior to offering HTC.	None	None	None	None	2	4,913	N/R	N/R
Molesworth [68]	2010	September 2007–October 2008	OS (Karonga, Malawi)	Africa	Door-to-door	A home-based cross-sectional survey of HIV in adults 15 y and older in northern Malawi was performed to assess the performance of rapid HIV antibody tests.	None	None	None	People who tested HIV-positive were referred to local clinical services.	1 (more to confirm if test positive or inconclusive or	16,894	N/R	N/R

											discordant)			
Mutale [69]	2010	2003	OS (Zambia)	Africa	Door-to-door	Home-based VCT was offerred to adults who expressed willingness to receive this in a population-based HIV survey.	None	None	None	None	2 (within following day for urban, 1–2 wk for rural)	5,012	2,936	58.58%
Naik [70]	2012	2009–2011	OS (KwaZulu-Natal, South Africa)	Africa	Door-to-door	Lay counsellors systematically visited all unique households in the designated HTC intervention clusters. After seeking permission from the household head, they offered free HIV testing to all household members aged 18 y and older. Those aged 14–17 y were also offered testing provided they had parental or guardian consent.	None	All clients were given a T-shirt stating "I know my status."	TB screening, STD screening, and family planning.	Referral letter to obtain a CD4 count and other HIV-related services at a local healthcare facility of their choice.	1	5,086	3,750	73.73%
Negin [71]	2009	2008	OS (Kenya)	Africa	Door-to-door	Home-based VCT was conducted in a high-prevalence area of Kenya. Cost analysis and other qualitative and quantitative data were collected.	Community meetings to spread awareness of testing services.	None	None	Referral to the nearest care and support services.	1	1,984	1,137	57.31%
Nuwaha [139]	2012	2004–2007	OS (Bushenyi, Uganda)	Africa	Door-to-door	Post-test survey based on door-to-door testing described in Tumwesigye et al. [74].	Education visits to local communities and mass media (radio) use.	None	Psychosocial support groups and referral to prevent social harm such as marital breakdown, domestic violence, and neglect.	Referral to local care providers offering basic preventative care, palliative care, and ART.	1	N/R	N/R	N/R
Sekandi [72]	2011	January–June 2009	OS (Kamapala, Uganda)	Africa	Door-to-door	Home-based HTC and a survey were offered to people 15 y and older in Kampala. Testing was performed by trained nurse-counsellors using national standard guidelines.	None	None	None	Referral to HIV care centres of their preference.	1	408	309	75.74%
Shapiro [30]	2012	February–September 2009	OS (North West Province, South Africa)	Africa	Door-to-door	Randomly selected houses were approached by a nurse and lay counsellors and offered HIV testing.	None	None	Symptom screening (cough, fever, night sweats, and/or weight loss) and sputum assessment for TB.	Appointments and referrals to the ART clinic for participants with HIV and CD4 counts < 250 cells/μl.	1	983	569	57.88%
Shisana [73]	2004	2002	OS (South Africa)	Africa	Door-to-door	Households were first notified about HIV testing survey. After several months retired and registered nurses were used as fieldworkers to provide HIV testing and to survey opinions and perceptions on HIV.	None	None	None	None	2	8,840	N/R	N/R
Tumwesigye [74]	2010	September 2004–March 2007	OS (Bushenyi District, Uganda)	Africa	Door-to-door	Home-based HTC was implemented in the Bushenyi District in Uganda. HTC was offered to all people over 14 y and to children at risk (i.e., mother with HIV or with unknown HIV status).	Sensitisation meetings were held in every parish. Informal information was handed out at informal gatherings. A weekly talk show on	None	HIV-infected individuals who had been coughing for 2 wk and/or were chronically sick were referred to health units for TB diagnosis, treatment of opportunistic infections, and evaluation for ART.	HIV-positive patients were referred for assessment for ART.	1	264,966	141,465	53.39%

							a local radio station was organised.							
Uwimana [75]	2012	March–December 2010	OS (KwaZulu-Natal, South Africa)	Africa	Door-to-door	Community care workers were trained to partake in TB/HIV/prevention of mother-to-child transmission activities and home-based HTC. Home-based HTC was performed in three villages.	None	None	Community care workers screened for TB and STDs.	HIV-positive individuals were referred for further care.	1	4,038	2,688	66.57% (74.47%)
van Rooyen [76]	2012	2011	OS (KwaZulu-Natal, South Africa)	Africa	Door-to-door	HTC conducted with follow-up of HIV-positive individuals at 1, 3, and 6 mo, along with risk reduction counselling.	None	None	HIV, TB, STD symptoms assessed for HIV-positive individuals.	Linked HIV-positive individuals to care and ART with follow-up.	N/R	673	451	67.01%
Vreeman [77]	2010	2008	OS (western Kenya)	Africa	Door-to-door	Home-based HTC of children between 18 mo and 12 y old. Children were included in the study if their mother was known to be dead, her living status was unknown, she was known to be infected with HIV, or her HIV serostatus was unknown.	Study team conducted community focus group discussions and worked with community leaders to prepare for visits.	None	None	None	1	1,294	637	49.23% (46.40%)
Wachira [127]	2012	August 2008–April 2010	OS (Rift Valley, Kenya)	Africa	Door-to-door	Retrospective study comparing home-based HTC with other testing methods. (The comparator arm consisted of a group that tested in a provider-initiated setting.)	None	None	None	Not within the study itself, but TB and HIV treatment included in the programme that the study observes.	N/R	946	678	71.67% (62.22%)
Wawer [78]	1998	December 1994–mid 1998	OS (western Kenya)	Africa	Door-to-door	Visits every 10 mo for testing and interventional (antibiotics) or control treatment (anti-helminthics with iron-folate supplements) offered to residents of study communities regardless of HIV status.	None	Participants offered a free bar of soap.	STD treatment.	HIV and STD treatment given as medicine or placebo.	N/R	11,618	6,423	55.28%
Welz [79]	2007	2003–2004	OS (Umkhanyakude district, South Africa)	Africa	Door-to-door	Trained fieldworkers visited all eligible individuals at home and obtained consent before testing household members for HIV.	None	None	None	None	2	11,551	6,859	59.38%
Wolff [81]	2005	2001	OS (15 villages in rural southwestern Uganda)	Africa	Door-to-door	Retrospective analysis of data from serosurvey data by local door-to-door and facility-based HTC efforts.	None	None	None	None	2	464	268	57.76%
Ahmed [27]	2013	2005–2010	OS (Niger, Kaduna, Nassarawa, and Benue States and the Federal Capital Territory, Nigeria)	Africa	Mobile	Mobile HTC was conducted in various locations in Nigeria. Several at risk populations were specifically targeted such as brothel and non-brothel sex workers and motorcycle taxi drivers. These groups were identified by informants who provided insight into community needs for HTC. (Facility-based HTC was performed at Asokoro District hospital.)	None	None	None	None	2 (May 2005–July 2006); 1 (August 2006–March 2010)	10,999	N/R	N/R
Bahwere [50]	2008	December 2002–May 2005	OS (Dowa District, Malawi)	Africa	Mobile	Community-based nutritional support programme offering HTC to graduates of the programme and current children with severe	None	None	Distribution of ready-to-use therapeutic foods for severe acute malnutrition.	Referred to paediatric and adult HIV care.	1 (2 if children <12 mo)	1,888	948	50.21%

						acute malnutrition.							
Chamie [51]	2012	2011	OS (Uganda)	Africa	Mobile	5-d multi-disease health campaign in rural Ugandan community.	Church and mosque announcements, distribution of posters, and radio announcements.	Transport stipend.	Screening for malaria, TB, hypertension, and diabetes.	Routine referrals to care for CD4 > 100 cells/µl. Enhanced linkage if CD4 > 100/µl.	1	4,343	2,666	61.39%
Chirawu [52]	2010	2003 and 2007	OS (four provinces in Zimbabwe)	Africa	Mobile	HTC offered after population-based survey for HIV prevalence (Regai Dzive Shiri Trial).	None	None	None	Participants referred to ongoing HIV care and support.	1	1,368	855	62.50%
Darling [53]	2012	2008–2010	OS (Lausanne, Switzerland)	Europe	Mobile	Clients of FSW were asked to complete a survey regarding their demographic information, their perceived HIV risk, and their HIV testing history. The participants were given a rapid finger stick HIV test at a van parked on site.	None	None	Clients were invited to fill out a second survey regarding condom use and STD testing history.	Positive tests were linked to confirmatory testing and medical care.	1	151	N/R	N/R
Edgil [136]	2011	2011	CE model (Swaziland)	Africa	Mobile	Male circumcision campaign including mobile HTC.	None	None	Male circumcision, STD treatment.	None	1	N/R	N/R	N/R
Grabbe [107]	2010	May 2005–April 2006	OS, CE model (Kenya)	Africa	Mobile	Mobile testing in schools, churches, and trucks. HTC at a stand-alone clinic as a control.	Posters advertising dates and locations of HTC were posted throughout host community in advance, local community mobilisation undertaken to encourage uptake.	None	None	None	1	47,539	20,128	42.34%
Granich [125]	2012	September 2009	OS (Nyanza, Kenya)	Africa	Mobile	3-d campaign using mobile HTC (as part of a multi-disease campaign for diarrheal diseases, malaria, and HIV).	Village "baraza" forums with local chiefs, radio and print messaging, and town cries with mobile trucks.	None	Provision of 60 condoms, an insecticide-treated bed net, a household water filter, and a 3-mo supply of cotrimoxazole for participants testing positive for HIV.	Visit from person with HIV to guide persons diagnosed with HIV to linkage.	1	5,198	3,108	59.79%
Govindasamy [124]	2011	August 2008–December 2009	OS (Cape Metropolitan Region, South Africa)	Africa	Mobile	Nurse-run, counsellor-supported mobile screening van testing for HIV, hypertension, diabetes, and TB. Visited shopping centres, taxi ranks and stations, and the roadside.	None	None	Screening for diabetes, hypertension, obesity, and TB.	Three telephonic attempts were made to track the participant, and if no contact number was available, a home visit was conducted.	1	192	116	60.42%
Hood [119]	2012	January–June 2007	OS (Botswana)	Africa	Mobile	HTC was provided in tents in a variety of settings, including outlying villages, entertainment events, transportation centres, and occupational settings.	None	None	None	None	1	21,237	10,931	52.41% (52.56%)

						Records of clients older than 18 y were analysed for this study. The comparator was clinics.							
Kahn [131]	2011	August–September 2009	CE model (Lurambi, Kenya)	Africa	Mobile	Describes costs of Lugada multi-disease campaign.	Ongoing programme of health education and community mobilisation conducted in the month before the campaign and during the campaign.	None	Provision of 60 condoms, an insecticide-treated bed net, a household water filter, and a 3-mo supply of cotrimoxazole for participants testing positive for HIV.	Visit from person with HIV to guide persons diagnosed with HIV to linkage.	1	N/R	N/R	N/R
Kawichai [54]	2007	September 2002–May 2003	OS (Chiang Mai Province, Thailand)	Asia	Mobile	Mobile testing in schools, temples, and health stations.	HIV/AIDS education was launched in each community 2–3 d prior to offering HTC, along with flyers and brochures and publicity from community leaders.	None	None	None	1	427	224	52.56% (54.00%)
Kranzer [55]	2012	September–November 2010	OS (Cape Metropolitan Region, South Africa)	Africa	Mobile	Mobile HTC was provided at a township shopping centre or a parking lot in front of the primary school. In addition to random participants electing to receive HTC at the mobile site, participants in a previous house-to-house seroprevalence survey were invited to mobile testing sites.	Community awareness was raised before and during the survey through pamphlets and meetings with the community advisory board and church women's groups.	70-rand vouchers redeemable at supermarkets were offered to some participants.	Screening for TB, diabetes, hypertension, and obesity.	None provided by study, but linkage was surveyed in population after testing.	1	1,813	834	46.00%
Lahuerta [108]	2011	February 2006–May 2009	OS (Escuintla, Guatemala)	Central and South America	Mobile	FSWs, MSM, transgender individuals, and people not reporting being a member of a risk group were offered HIV and syphilis rapid tests and were interviewed about their sociodemographic and risk behaviour.	None	None	Syphilis testing and condom provision.	Referral to a treatment programme.	1	513	319	56.66% (54.49%)
Larson [126]	2012	2010	OS (Johannesburg, South Africa)	Africa	Mobile	A mobile HTC programme around Johannesburg piloted the integration of point-of-care CD4	None	None	None	Referral to an HIV care and treatment site.	1	311	194	62.40%

						testing, using the Pima analyser, to improve linkages to HIV care.								
Lugada [56]	2010	16–22 September 2008	OS (Lurambi, Kenya)	Africa	Mobile	1-wk campaign including mobile HTC at designated sites with a mobile truck, in addition to support sites during times of high demand.	Ongoing programme of health education and community mobilisation conducted in the month before the campaign and during the campaign.	None	Provision of 60 condoms, an insecticide-treated bed net, a household water filter (women), an individual water filter (men), and a 3-mo supply of cotrimoxazole for participants testing HIV-positive.	Referral to appropriate care, free 3-mo supply of cotrimoxazole prophylaxis.	1	47,173	28,906	61.28%
McCoy [120]	2013	2011–2012	OS (Oakland, California, US)	North America	Mobile	Participants were tested for HIV infection and asked to refer up to three others. Testing was at storefront offices and in mobile units. Different incentive schemes were evaluated.	None	Either a flat incentive (US$20) for eligible recruits or conditional incentives (US$10–US$35) for eligible recruits in priority groups, such as first-time testers.	None	Participants found to have HIV were referred for confirmatory testing and linked into care.	1	291	105	36.10%
Maheswaran [28]	2012	February–June 2009	OS (KwaZulu-Natal, South Africa)	Africa	Mobile	The mobile testing unit had capacity to provide HTC for up to six clients at any one time in either a truck or in additional tents. The facility was set up at local community venues, chosen for convenience to the community and access to the greatest number of clients as possible, often near schools or shops. All participants were provided with educational leaflets and condoms.	Before implementation, community mobilisation and education was achieved through a series of meetings with local political and traditional leaders, and distribution of information leaflets and road shows to market the service.	None	None	Participants testing positive for HIV were provided with referral letters to the local HIV clinic for further follow-up.	1	1,013	601	59.33%
Mahler [57]	2011	21 June–31 July 2010	OS (Iringa, Tanzania)	Africa	Mobile	HTC offered during a government-run campaign for voluntary male circumcision.	Traditional theatre, small group sessions, one-to-one peer education, radio	None	Circumcision, clients with STDs were referred to STD treatment centres at the same health facility.	HIV-positive clients were referred to HIV care and treatment services at each circumcising site.	1	10,252	N/A (male circumcision campaign)	N/A (male circumcision campaign)

							advertisements, regional leaders promoting the campaign, and speeches to community groups.						
Morin [109]	2006	March 2002–August 2003	OS (Epworth and Seke, Zimbabwe)	Africa	Mobile	A mobile van provided free, anonymous HTC using two parallel rapid HIV tests, visiting the sites on a rotating basis four times for 4 d per site. HTC services as well as HIV/AIDS educational materials and condoms were made available.	Information pamphlets distributed at markets, posted advertisements at local HIV post-test clubs.	Participants in study were paid US$5.	No	No	1	1,099	458	41.67%
Nglazi [110]	2012	August 2008–August 2010	OS (Cape Metropolitan Region, South Africa)	Africa	Mobile	A modified van with two testing rooms, a counselling room, and a bathroom. The intervention consisted of a group that was given incentives for HTC.	One male recruiter invited unemployed men to attend the mobile HTC service on a predetermined day and venue.	80-rand food voucher provided to unemployed or casually employed men.	Screening for diabetes, hypertension, obesity, and STDs.	Referral to their local healthcare facility or healthcare provider. Those with CD4 < 200 cells/μl were referred to ART specifically.	1	8708	N/A (focused on testing unemployed men)	N/A (focused on testing unemployed men)
Ostermann [58]	2011	December 2007–April 2008	OS (Kilimanjaro Region, Tanzania)	Africa	Mobile	Mobile HTC was offered for 2 to 3 wk in up to two locations per village.	Campaigns were advertised at ward leaders' offices, churches, and other key places, as well as via bullhorn advertising on the 2 d preceding and the first day of testing at each location.	None	None	None	1	883	423	47.90%
Slesak 1 [26]	2012	2006	OS (Luang Namtha Province, Lao People's Democratic Republic)	Asia	Mobile	Assessed the HIV vulnerability in four minority villages alongside the new road in Luang Namtha Province using structured interviews VCT for HIV.	None	None	None	None	1	924	N/R	N/R
Slesak 2 [26]	2012	2008	OS (Luang Namtha Province, Lao People's Democratic Republic)	Asia	Mobile	Same as Slesak 1 [26] above	None	None	None	None	1	538	N/R	N/R
Spielberg [111]	2011	April 2001–April 2002	OS (Washington state, US)	North America	Mobile	HTC was offered to people of colour at bars and parks where people at high risk for HIV	Mass media campaigns, incentives,	US$10 incentives were offered	HIV/STD risk reduction counselling.	None	1 or 2	595	179	30.08%

						congregate. Data were compared with health-department-based testing.	and word of mouth.	for testing.						
Sweat [23]	2011	March 2006–April 2009	RCT (ten communities in Tanzania)	Africa	Mobile	Communities were paired and randomly placed in a group with community-based HTC and standard clinic-based HTC or a group with only clinic-based HTC. In community-based HTC sites, intervention consisted of deployment of a mobile VCT unit comprising VCT counsellors, a nurse-counsellor, and an outreach worker/driver.	Educating communities about HIV and encouraging discussion in the community with the intent to increase awareness, decrease stigma, and encourage people to consider undergoing HIV testing.	None	None	None	1	2,341	976	41.69% (47.15%)
Sweat [23]	2011	January 2006–July 2009	RCT (14 communities in Thailand)	Asia	Mobile	Same as Sweat [23] above.	Same as Sweat [23] above.	None	None	None	1	9,361	5,102	54.50% (65.64%)
Sweat [23]	2011	January 2006–July 2009	RCT (Eight communities in Zimbabwe)	Africa	Mobile	Same as Sweat [23] above.	Same as Sweat [23] above.	None	None	None	1	5,437	2,621	48.21% (46.01%)
van Rooyen [59]	2012	2005	OS (South Africa)	Africa	Mobile	At each site a multi-disciplinary team consisting of a team leader, three trained VCT counsellors, a nurse, and a driver/community outreach worker delivered the service. A mobile caravan equipped with a laboratory and counselling spaces was set up at visible, convenient community venues such as markets, churches, community and shopping centres, and transportation hubs (e.g., bus and taxi stands).	Extensive consultations with traditional and political leadership, health and social services, and non-governmental and community-based organisations. Distribution of flyers and brochures in public spaces.	None	None	Clients were given the option of receiving HIV test certificates documenting their HIV status should they require this to access other treatment and healthcare services.	1	988	434	44.00%
Wringe [129]	2012	2006–2008	OS (Karonga, Malawi; Kisesa, Tanzania; Masaka, Uganda; and Manicaland, Zimbabwe)	Africa	Mobile	Data on VCT and ART use among HIV-infected persons were analysed from Karonga (Malawi), Kisesa (Tanzania), Masaka (Uganda), and Manicaland (Zimbabwe), where free ART provision started between 2004 and 2007. ART coverage was compared across sites by calculating the proportion on ART among those estimated to need treatment, by age, sex, and educational attainment.	None	None	None	Referral to clinic for two of the four study programmes.	N/R	N/R	N/R	N/R

Terris-Prestholt [135]	2006	1996–1999 (cost data is in 2001 US dollars)	CE model (Masaka, Uganda)	Africa	Mobile	Two trained counsellors visited communities to provide HTC.	General community meetings in conjunction with dramas, video shows, education via community-based volunteers.	None	Condom provision and STD treatment.	None	1	4,425	N/R	N/R
Truong [112]	2011	March 2002–August 2003	OS (Mashonaland East Province and Greater Harare, Zimbabwe)	Africa	Mobile	Four HIV nurse-counsellors provided HTC from a modified caravan.	Pamphlets distributed describing VCT services and the study.	None	None	None	1	1,096	456	41.61%
van Schaik [121]	2010	August 2008–December 2008	OS (Cape Metropolitan Region, South Africa)	Africa	Mobile	Nurse-run, counsellor-supported mobile screening van. Visited shopping centres, taxi ranks and stations, and the roadside.	None	None	Screening for diabetes, hypertension, and obesity.	None	1	2,499	1,209	48.38% (60.11%)
Ahmed [27]	2013	2005–2010	OS (Niger, Kaduna, Nassarawa, and Benue States and the Federal Capital Territory, Nigeria)	Africa	Key population (FSW)	Mobile HTC was conducted in various locations in Nigeria. Several at risk populations were specifically targeted such as brothel and non-brothel sex workers and motorcycle taxi drivers. These groups were identified by informants who provided insight into community needs for HTC. (Facility-based HTC was performed at Asokoro District hospital.)	None	None	None	None	2 (May 2005–July 2006); 1 (August 2006–March 2010)	1,590	N/A	N/A
Balaji [94]	2013	2008	OS (22 cities in the US)	North America	Key population (MSM)	MSM who responded to the National HIV Behavioral Surveillance System were included in a study that assessed incidence density, prevalence, and risk factors associated with HIV infection in the US.	None	None	None	None	1 or 2 depending on testing site	9,342	N/A	N/A
Bungay [114]	2013	2006–2009	OS (western Canada	North America	Key population (FSW)	Community health workers visited sex venues that were previously identified. FSW received health education, health and social service (e.g., immigration, legal, income assistance) referrals and accompaniment to appointments, condom and lubricant distribution, and point-of-care HIV testing. They also completed a sociodemographic survey that included sex work characteristics and HIV testing history.	None	None	Self-collected vaginal swabs for human papillomavirus, chlamydia, and gonorrhea.	None	1	129	N/A	N/A
Bell [102]	2003	1997–2000	OS (Illinois and New Jersey, US)	North America	Key population (adolescents)	HTC was offered at youth-oriented community agencies and through mobile vans that travelled to areas where adolescents often congregate. .	Social events and psychodram a presentation s to adolescents	None	None	None	2	4,224	1,581	37.43%

							and to distribute HIV prevention information and materials to them.						
Bingham [113]	2008	May 2001–December 2002	OS (Los Angeles, California, US)	North America	Key population (MSM)	HTC was offered onsite at bathhouses on weekdays, weekends, and nights during four to seven periods each week (for a minimum of a total of 28 h). Demographic and behavioural data were collected from the participants.	Men were recruited via announcements on the bathhouse's public announcement system, word of mouth, and flyers posted with the bathhouse.	None	None	None	2	458	N/A	N/A
Bowles [106]	2008	2004–2006	OS (Boston, Chicago, Detroit, Kansas City, Los Angeles, San Francisco, and Washington, D.C., US)	North America	Key population (MSM and PWID)	Community-based organisations provided HTC in mobile testing units to reach populations at high risk of HIV acquisition and less likely to access traditional HTC.	None	Some sites provided snacks, hats, gloves, or grocery gift cards.	None	People with confirmed HIV infection were referred to care, treatment, prevention, and support services.	2	2,585	N/R	N/R
Bucher [105]	2007	2003–2004	OS (San Francisco, California, US)	North America	Key population (MSM, PWID, and FSW)	Teams of four to six staff members visited key population venues and approached individuals and provided appointment cards for onsite testing.	None	Participants received US$15 for rapid testing and US$15 upon return for confirmatory results.	None	Referral to primary healthcare providers was made after confirmatory visit.	2	1,213	259	21.35%
CDC [98]	1997	July–September 1995	OS (Colorado Springs, Colorado, US)	North America	Key population (PWID)	Two nurses visited community organisations, public parks, and street intersections to offer HTC to people who they believed were at high risk. Demographic and HIV behavioural information was collected from the participants.	None	None	None	None	2	212	74	34.91%
CDC [93]	2007	2004–2006	OS (Detroit, Baltimore, Jackson, Charlotte, St. Louis, Washington, D.C., Oakland, San Francisco, and Chicago, US)	North America	Key population (MSM)	Community-based organisations provided HTC at gay pride events attended primarily by MSM from racial/ethnic minority groups.	None	Nonmonetary incentives valued at less than US$10 were sometimes offered to increase participation.	None	None	2	133	N/A	N/A
Champenois [115]	2012	February 2009–June 2010	OS (Montpellier, Lille, Bordeaux, and Paris, France)	Europe	Key population (MSM)	HIV tests were offered during 3-h sessions once or twice a week in the evening and/or on the weekend by trained counsellors working for a local community-based organisation. MSM were specifically sought out with the use of a targeted advertisement campaign.	Communication campaigns (posters, flyers, web banners, and ads) at commercial and non-commercial	None	None	Participants who tested positive for HIV were referred to HIV clinics for confirmatory blood tests and linkage to care.	1	532	N/A	N/A

							gay venues, as well as in gay websites, magazines, and organisation s.							
DiFranceisco [116]	1998	January 1992–June 1995	OS (Wisconsin, US)	North America	Key population (MSM and PWID)	Outreach services including HTC were provided via several different settings including bars, gay pride parades, and community health fairs.	None	None	None	None	2	12,171	5,271	43.30%
Galvan [95]	2006	N/R	OS (Los Angeles, California, US)	North America	Key population (MSM)	Counsellors offered HTC to every third person attending bars frequented by MSM. Behavioural surveys were also conducted.	None	US$15 was provided at the end of their tests.	Screening for alcohol and drug dependence, depression, syphilis, gonorrhea, and chlamydia.	Referrals for follow-up mental health or substance abuse counselling and medical care as necessary.	1	343	N/A	N/A
Gelberg [99]	2012	June 2003–February 2004	OS (Los Angeles, California, United States)	North America	Key population (PWID)	A homeless population was sampled from 41 homeless shelters and meal programmes in the Skid Row area of downtown Los Angeles. Participants were interviewed and tested for hepatitis C, HIV, and hepatitis B. A group of PWID were analysed for the purposes of this paper.	None	None	Pariticipants were tested for hepatitis C and hepatitis B.	None	2	92	8	8.70%
Keenan [117]	2001	April 1999–September 2000	OS (Minneapolis, Minnesota, US)	North America	Key population (MSM, PWID, and FSW)	Onsite HTC was offered at chemical dependency programmes, homeless shelters, a needle exchange programme, an organisation for MSM, and a support group for FSW.	Contacted community-based organisation s to find a client base and encourage support for HTC.	None	None	None	1	735	233	31.70%
Lahuerta [108]	2011	February 2006–May 2009	OS (Escuintla, Guatemala)	Central and South America	Key population (MSM)	A mobile van offering HTC travelled to commercial sex venues and sites frequented by MSM and transgender individuals (including bars, brothels, MSM hair salons, and streets where sex workers met clients to obtain the permission of their owners/managers).	None	None	Syphilis testing and condom provision.	Participants with a positive confirmatory HIV test were referred to a treatment programme.	1	385	N/A	N/A
Lahuerta [108]	2011	February 2006–May 2009	OS (Escuintla, Guatemala)	Central and South America	Key population (FSW)	Same as Lahuerta [108] above.	None	None	Syphilis testing and condom provision.	Same as Lahuerta [108] above.	1	438	N/A	N/A
Liang [104]	2005	May 2003–February 2004	OS (Baltimore, Maryland, US)	North America	Key population (FSW and PWID)	A mobile van staffed with a nurse practitioner, phlebotomist, community health educators, and outreach workers was stationed in areas of high STD morbidity, drug use, and commercial sex work.	Participants recruited from street by outreach workers.	None	None	None	1 or 2	440	168	38.18%
Lister [90]	2005	October 2002–September 2003	OS (Melbourne, Australia)	Australia	Key population (MSM)	Nurses offered STD screening, including HTC, to patrons at MSM saunas.	Minimal promotion with business-sized cards,	None	Testing for gonorrhea, chlamydia, hepatitis A and B, HIV, and syphilis.	All men who tested positive for STDs were followed up.	2	161	N/A	N/A

							one or two small posters on display in the saunas, and occasional advertising in the local gay press.							
Nhurod [101]	2010	December 2005	OS (Bangkok, Thailand)	Asia	Key population (FSW)	Mobile HTC was offered as part of World AIDS Day. FSW were specifically sought out and tested.	Public announcements of the availability of free HIV and syphilis testing in the outreach area and invitations to interested street-based sex workers.	Transportation costs reimbursed.	VCT was offered for HIV and syphilis.	None	2	81	N/A	N/A
Outlaw [91]	2010	2006–2008	OS (Detroit, Michigan, US)	North America	Key population (MSM)	HTC was offered at community venues for African American MSM between the ages of 18 and 26 y. Counselling sessions were either motivational or traditional.	None	Participants received US$20.	None	None	2	47	N/A	N/A
Robbins [103]	2010	May–December 2008	OS (Odesa, Kyiv, and Donetsk, Ukraine)	Europe	Key population (adolescents)	Mobile vans were used to offer HTC in locations where street youth congregate (e.g., near fast-food restaurants, recreation areas, computer clubs, and metro stops) in various locations in the Ukraine.	None	Food, juice, or clothing was offered to participants completing HTC.	None	Referred to medical care or case management by programme staff.	1	929	223	24.00%
Shrestha [122]	2008	April 2004–March 2006	CE model (Kansas City, Missouri, and Detroit, Michigan, US)	North America	Key population (MSM, PWID, and FSW)	A mobile van provided HTC in health fairs, public parks, homeless shelters, substance abuse and mental health treatment centres, soup kitchens, motels, bars and nightclubs, areas frequented by commercial sex workers, street corners, needle-exchange programmes, and bathhouses/bars used by MSM.	None	Transportation tokens or grocery vouchers.	None	None	1	N/R	N/R	N/R
Shrestha [133]	2010	October 2003–December 2005	CE model (Boston, Philadelphia, and Washington, D.C., US)	North America	Key population (MSM and PWID)	Counsellors were enlisted and trained to test members of their social network who were at high risk of acquiring HIV infection.	None	Gift cards ranging from US$6.83 to US$18.53 were provided to counsellors (when identified and for each person they tested) and gift cards from US$5.43 to US$11.22 were provided to people who completed testing.	None	None	1	N/R	N/R	N/R

Shrestha [134]	2011	April 2005–December 2006	CE model (New York City, New York, and San Francisco, California, US)	North America	Key population (PWID and transgender)	Mobile vans were used to offer HTC in bars and night clubs frequented by transgender persons. Transgender persons were also asked to refer other people who are at high risk of HIV infection (sexual partners, transgender peers, and drug-using partners). Demographic information of the participants was obtained. The cost of the testing programme was analysed.	None	The programmes provided cash, food, beverages, donated items, cash (US$20 in New York City), gift cards (US$25 in San Francisco for testing and US$10 for referring people at high risk of HIV infection), or products (bags, magazines, or cosmetics) to participants who completed surveys and HIV testing.	None	None	1	N/R	N/R	N/R
Smith [96]	2006	1999–2001	OS (Los Angeles, California, US)	North America	Key population (MSM)	HIV counsellors asked clients at an MSM community centre whether they were interested in rapid HIV testing. Conventional testing was used to confirm positive results.	None	None	None	None	2	1,201	N/A	N/A
Spielberg [92]	2005	1999–2000	OS (Seattle, Washington, US)	North America	Key population (MSM and PWID)	HTC was offered to people at bathhouses and needle exchange sites. Respondents 14 y or older who spoke English were eligible for HTC.	None	None	None	None	1 or 2	761	94	12.35%
Stein [118]	2011	August 2005–July 2006	OS (Houston, Miami, New York City, Newark, and San Juan, US)	North America	Key population (MSM)	HTC was offered in at high-risk venues, such as bars and parks where people at high risk for HIV congregate.	Community-based organisations used mass media campaigns to reach clients.	Initially no incentives were given, but over time US$10 was offered for HIV testing.	Starting in 2006 the programme used the Computer Assessment and Risk Reduction Education tool for routine use with rapid HTC. This computer tool gave patients HIV/STD risk reduction counselling.	Clients with a positive HIV test result were referred to medical care.	1	1,723	N/A	N/A
Sy [97]	1998	1997	OS (South Carolina, US)	North America	Key population (MSM)	Health department staff provided HTC in bars frequented by MSM.	None	Condoms, lubricants, and T-shirts.	None	None	2	64	N/A	N/A
Yin [123]	2012	2009–2010	OS (Shenzhen, Guangzhou, and Changzhou, China)	Asia	Key population (MSM)	MSM participants were recruited from different settings, including an STD clinic in Shenzhen, a health centre in Guangzhou, and several MSM venues in Changzhou. After informed consent was obtained, the men were asked to provide blood samples and were interviewed by outreach workers, using a structured questionnaire consisting of questions related to	None	None	Screening for herpes simplex virus 2.	None	2	1,462	N/A	N/A

						demographic and behavioural characteristics.							
Choko [47]	2011	March–July 2010	OS (Blantyre, Malawi)	Africa	Self-testing	Members from 15 randomly selected houses and randomly selected community peer groups (such as sports teams, micro-finance, and church groups) were offered the option to receive no testing, HTC, or self-testing with confirmatory HTC.	None	None	None	Written referral into HIV care services.	1	283	147	51.94%
Frank [48]	1997	N/R	OS (nine cities in US: Cincinnati, Lawrenceville, Mogadore, Portland, West Orange, Fresno, New Orleans, Philadelphia, and San Francisco)	North America	Self-testing	Participants were given home collection kits to obtain their own blood samples by finger prick. These samples were tested and compared with both a finger prick and venous blood sample collected by a phlebotomist.	Recruitment was performed using radio and newspaper advertisements offering a free HIV test.	Up to US$25 payment for participation.	None	None	1 (results given by call)	1,255	471	37.53%
Spielberg [49]	2000	July–November 2006	OS (Denver, Philadelphia, Providence, and San Francisco, US)	North America	Self-testing	Staff taught participants to collect oral fluids and dried blood spots. Participants then collected their own specimens routinely for HTC.	None	US$10 was given at enrolment, at each of the three required specimen collection events, for completing the end-of-study questionnaire, and for completing all required specimen and data collection (US$60 total).	None	None	2	241	48	19.92% (17.14%)
Corbett [22]	2006	2002–2004	RCT (Harare, Zimbabwe)	Africa	Workplace	22 small- and medium-sized businesses were randomly allocated to either onsite HTC or referral to a fixed HTC site (the latter participants were given a voucher for offsite testing and a 2-wk appointment to discuss the results).	None	None	Isoniazid preventive therapy for participants with HIV without active TB.	Employees with HIV were offered isoniazid preventive therapy (if TB skin test positive and without active TB) and/or cotrimoxazole (if WHO stage 2–4 HIV). ART referral was not part of the package of care offered to participants testing positive for HIV.	1	3,845	431	11.21% (13.10%)
Feeley [82]	2007	September 2001–January 2005	OS (Kigali and Gisenyi, Rwanda)	Africa	Workplace	Free HTC and lifelong ART were offered to all employees of Heineken Rwanda and their immediate dependents, including children.	Education initiative, community leader testimony, and kickoff of national HTC and ART programme.	None	None	ART provided to those with CD4 counts < 300 cells/μl.	1	634	N/R	N/R

Kwena [83]	2013	2012	OS (Kisumu County, Kenya)	Africa	Workplace	A cross-sectional survey of married couples was performed in fishing communities in Lake Victoria, Kenya. The couples were interviewed about a variety of details including marital and sexual relationships, migratory factors, and demographic details. Couples were given HTC with rapid tests.	None	None	None	None	1	1,090	545	50.00%
Machekano [84]	1998	March 1993–June 1995	OS (Harare, Zimbabwe)	Africa	Workplace	HTC offered to males from 40 factories in Harare. Serological tests and interviews were conducted every 6 mo. Testing took place at an offsite location that was located within a 30-min drive of each of the factory sites.	None	Participants were reimbursed for transportation to the project clinic, which was located within a 30-min drive of all work sites.	Screening for other STDs.	None	2	2,060	N/A (only men were employed at workplace)	N/A (only men were employed at workplace)
Todd [85]	2012	2010–2011	OS (Afghanistan)	Asia	Workplace	Potential Afghan National Army recruits were offered medical screening and basic training exercises at Kabul Military Training Center.	None	None	Testing for syphilis, herpes simplex 2 virus, and hepatitis C virus.	None	2	None	N/R	N/R
Van der Borght [86]	2010	September 2001–December 2007	OS (Democratic Republic of the Congo, Rwanda, Burundi, Congo, and Nigeria)	Africa	Workplace	Heineken started a comprehensive HIV workplace programme, including ART, for all employees (current and retired) and their families. Voluntary and confidential HTC was completed on site by physicians and nurses of the company’s health services.	Information and education campaigns among employees (adapted to local conditions, resources, and expertise) were regularly conducted. Peer groups of employees were also utilised to enhance HIV awareness among the employees.	None	Many of the tests occurred as a result of a hepatitis B vaccination campaign that offered VCT.	CD4 count was conducted immediately after someone tested positive for HIV to determine eligibility for ART.	1	9,723	3,865	39.75%
McConnel [132]	2005	June 2002–May 2003	CE model (KwaZulu-Natal, South Africa)	Africa	Church-based	Church-based HTC with rapid results offered to the general population.	None	None	None	None	N/R	N/R	N/R	N/R
Henry-Reid [87]	1998	January 1993–January 1994	OS (Chicago, Illinois, US)	North America	School-based	Adolescents who were sexually active, were pregnant, had a history of at least one STD, or had a history of substance abuse were seen in a school clinic in an inner-city high school and offered HTC by a health educator.	None	None	All adolescents received HIV/STD education as part of the pre-test and post-test counselling.	None	2	20	17	85.00% (74.60%)
Kharsany [88]	2012	September 2010–	OS (rural KwaZulu-Natal Midlands,	Africa	School	A cross-sectional, anonymous, linked survey was undertaken in	In preparation	None	None	All learners were provided with	2	1,566	852	55.72%

		February 2011	South Africa)			two randomly selected high schools in Vulindlela. A trained team of nurses and counsellors visited the two schools to provide information about HTC and to offer it to anyone who wanted to receive it.	for the survey, several consultative meetings were held with the Department of Education at the provincial, district, and school level.			information on how to access HIV testing and care services and HIV risk reduction counselling, including access to medical male circumcision for male learners.				
Naughton [128]	2011	June 2008–August 2009	OS (Eastern Cape, South Africa)	Africa	School-based	A comprehensive HIV service providing social support and HTC was provided in 12 schools.	None	None	None	Multiple follow-up attempts were made by phone and in person by trained lay counsellors, and a dedicated adolescent HIV support group with subsidised travel costs was created.	1	682	442	64.81%
Patel [89]	2012	2009	OS (Zimbabwe)	Africa	School-based	16 rural community-based, community-run play centres were established to provide health, nutritional, and psychosocial support for children aged 5 y and younger exposed to or living with HIV, coupled with family support groups for their families/caregivers.	Community sensitisation meetings with the support and assistance of local gatekeepers, chiefs, headmen, councillors, and the police. These meetings targeted parents and caregivers of children younger than 5 y of age.	None	Child attendance, family circumstances, age-appropriate weight for-height development, health status (including illness episodes), vaccinations, HIV exposure, and infection status were documented.	Every child who was diagnosed HIV-positive was enrolled in an opportunistic infections/ART programme and began taking prophylactic doses of cotrimoxazole.	1	410	N/R	N/R
